# Isolation of Phenolic Metabolites and Evaluation of the Antioxidant and Metabolic Enzyme-Inhibitory Activities of *Polygonum equisetiforme* Sm.

**DOI:** 10.3390/molecules31183327

**Published:** 2026-09-19

**Authors:** Merve Yüzbaşıoğlu Baran, Mehmet Uzun, Zekiye Ceren Arıtuluk Aydın, András Simon, Ayşe Kuruüzüm-Uz

**Affiliations:** 1 Department of Pharmacognosy, Gülhane Faculty of Pharmacy, University of Health Sciences, 06108 Ankara, Türkiye; 2 Department of Pharmacognosy, Faculty of Pharmacy, Hacettepe University, 06100 Ankara, Türkiye; mehmetuzun@hacettepe.edu.tr (M.U.); ayseuz@hacettepe.edu.tr (A.K.-U.); 3 Department of Pharmaceutical Botany, Faculty of Pharmacy, Hacettepe University, 06100 Ankara, Türkiye; zceren@hacettepe.edu.tr; 4 Department of Inorganic and Analytical Chemistry, Budapest University of Technology and Economics, 1111 Budapest, Hungary; simon.andras@vbk.bme.hu

**Keywords:** lipase inhibition, *Polygonum* spec., phenolics, antioxidants, metabolic enzyme inhibition

## Abstract

*Polygonum equisetiforme* Sm. is traditionally used in the Mediterranean region as a medicinal plant and herbal tea ingredient. This study aimed to characterize its phenolic constituents and evaluate its in vitro potential against metabolic targets associated with obesity and diabetes. Methanolic and defatted aqueous extracts of the aerial parts were prepared, and the aqueous extract was fractionated using chromatographic techniques. The isolated compounds were characterized by one- and two-dimensional NMR spectroscopy, supported by mass spectrometry where available, and compared with published spectroscopic data. Antioxidant activity was evaluated using 2,2-diphenyl-1-picrylhydrazyl (DPPH), superoxide anion radical (O_2_•^−^), nitric oxide (NO), cupric ion reducing antioxidant capacity (CUPRAC), ferric reducing antioxidant power (FRAP), and Trolox equivalent antioxidant capacity (TEAC) assays. Inhibitory effects against α-amylase, α-glucosidase, and pancreatic lipase were determined, and glucose uptake was assessed in 3T3-L1 cells. Seven phenolic compounds were identified: 3-*O*-acetyl-(−)-epicatechin, liquiritin, catechin, tamarixetin, quercetin, quercetin-3-O-glucoside, and quercetin-3-*O*-galactoside. Among these, 3-*O*-acetyl-(−)-epicatechin and tamarixetin are reported for the first time in the genus Polygonum. The phenolic-rich fractions exhibited pronounced antioxidant activity, with Fr. E showing the strongest DPPH radical-scavenging activity (IC_50_ = 6.00 µg/mL). The methanolic and aqueous extracts inhibited α-amylase (IC_50_ = 310.95 and 285.00 µg/mL), α-glucosidase (IC_50_ = 374.04 and 362.82 µg/mL), and pancreatic lipase (IC_50_ = 519.20 and 868.50 µg/mL), respectively. At 100 µg/mL, both extracts enhanced glucose uptake in 3T3-L1 cells, with a further significant increase observed in the presence of insulin. These findings expand the phytochemical knowledge of *P. equisetiforme* and demonstrate its multi-target in vitro potential against metabolic processes associated with obesity and diabetes.

## 1. Introduction

The genus *Polygonum* (Polygonaceae) is known worldwide for its rich phytochemical content and various ethnomedicinal applications. Species within this genus have traditionally been used to treat a range of illnesses including gastrointestinal disorders, skin diseases, respiratory conditions, and diabetes [1]. The genus *Polygonum* L. (Polygonaceae) includes more than 170 species, mostly distributed in northern temperate regions [2,3,4]. In Türkiye, the genus is represented by 41 species, of which 19 are endemic [5,6]. These plants are particularly rich in flavonoids, phenolic acids, stilbenes, and tannins [7,8], which contribute to their broad pharmacological potential. Pharmacological studies have reported antioxidant, antimicrobial, anti-inflammatory, anticancer, antiviral, lipid-lowering, neuroprotective, and antidiabetic effects in various *Polygonum* species [1]. Ethnobotanical records from Türkiye highlight the traditional use of *Polygonum cognatum* Meissn., locally known as “madımak,” which is consumed as a cooked herb, particularly in spring when its young shoots are harvested. In Turkish folk medicine, this species has been used for various purposes, most notably for its diuretic properties and in the management of diabetes mellitus [9]. *P. equisetiforme* has been traditionally used in North Africa and the Eastern Mediterranean for the treatment of sore throats, colds, and coughs [10]. The dried aerial parts are commonly added to herbal teas for their flavoring properties and general health-promoting effects [11,12]. Importantly, no serious toxicity has been reported for this species, supporting its continued use in traditional medicine and even as fodder for animals [1,8].

Preliminary phytochemical analyses indicate that *P. equisetiforme* is rich in polyphenols. Chemical profiling studies revealed high levels of total phenolics and flavonoids correlating with strong antioxidant activity. HPLC-DAD and LC-MS/MS analyses have identified flavonoids such as (+)-catechin, epicatechin, myricetin, quercetin glycosides (e.g., quercetin-3-*O*-galactoside, quercetin-3-*O*-rhamnoside), polymethoxylated flavones (cirsiliol, cirsilineol), and smaller amounts of flavanones like naringenin and its glycoside naringin, along with the flavone luteolin. Phenolic acids, including gallic acid, protocatechuic acid, and quinic acid, were also identified [7,8,13]. Also, quercetin, several quercetin glycosides, isorhamnetin, and betmidin have been isolated from this species [14,15].

Obesity, characterized by excessive accumulation of body fat, is a chronic metabolic disorder that poses a significant threat to global health. The increasing prevalence of obesity has led to its classification as an epidemic by the World Health Organization [16]. It is associated with several comorbidities, including type 2 diabetes, cardiovascular diseases, and certain types of cancer [17]. Its increasing prevalence has encouraged the investigation of different strategies targeting metabolic processes involved in lipid digestion and glucose homeostasis [18]. Among these strategies, plant-derived bioactive compounds have attracted attention because of their potential effects on obesity-related metabolic pathways. *P. equisetiforme*, with its diverse phytochemical profile including phenolic compounds and flavonoids, has potential relevance in this context. Previous studies have reported antioxidant activity and inhibitory effects of this species on enzymes such as α-amylase and α-glucosidase, suggesting potential effects on carbohydrate metabolism [13].

However, its effects on other metabolically relevant targets, including pancreatic lipase and cellular glucose uptake, remain insufficiently investigated. Furthermore, detailed isolation studies are required to identify the constituents potentially contributing to these activities.

α-amylase, pancreatic lipase, and α-glucosidase are key enzymes involved in carbohydrate and lipid metabolism, and their inhibition represents a potential approach for modulating postprandial glucose and lipid availability. α-glucosidase inhibitors delay carbohydrate absorption and glucose release in the intestines, while amylase inhibitors limit the breakdown of starches, both of which contribute to better glycemic control [19,20]. Similarly, lipase inhibitors reduce the hydrolysis and subsequent absorption of dietary lipids [21]. Additionally, the regulation of glucose uptake by adipocytes contributes to glucose homeostasis and cellular energy metabolism. The 3T3-L1 preadipocyte cell line is commonly used for in vitro studies on adipogenesis and glucose uptake, providing valuable insights into the effects of compounds **on** cellular glucose metabolism [22,23].

In this study, we aimed to isolate and characterize bioactive secondary metabolites from *P. equisetiforme* grown in Türkiye and to investigate their biological relevance through metabolic enzyme inhibition (α-amylase, α-glucosidase, and pancreatic lipase) and the modulation of glucose uptake in 3T3-L1 preadipocytes. Antioxidant assays (CUPRAC, FRAP, TEAC, SO, NO, and DPPH) were also performed to determine the antioxidant capacity of the isolated compounds, considering the association between oxidative stress and metabolic dysfunction [24]. Overall, this work provides a comprehensive analysis of the chemical composition and bioactivities of *P. equisetiforme*, highlighting its potential relevance to carbohydrate and lipid metabolism.

## 2. Results

### 2.1. Total Phenolic, Flavonoid, and Tannin Contents of Extracts and Fractions

Because Fr. A was eluted with water and was therefore expected to contain sugars and other highly polar non-phenolic constituents, it exhibited a relatively low total phenolic content (127.5 ± 4.2 mg GAE/g), while its total flavonoid and condensed tannin contents were below the detection limit. Fr. A also showed no measurable antioxidant activity; therefore, subsequent bioactivity evaluations focused primarily on the phenolic-rich fractions, Fr. B–G.

The crude extracts and fractions of *P. equisetiforme* contained substantial amounts of phenolic compounds (Table 1). PE-MeOH exhibited a slightly higher total phenolic content (TPC; 523 ± 8 mg GAE/g) than that of PE-H_2_O (463 ± 5 mg GAE/g). Among the fractions, Fr. E showed the highest TPC (835 ± 6 mg GAE/g), followed by Fr. D (799 ± 9 mg GAE/g) and Fr. G (760.6 ± 8.3 mg GAE/g).

The total flavonoid contents of PE-MeOH and PE-H_2_O were comparable, at 103.4 ± 2.5 and 104.0 ± 1.8 mg QE/g, respectively. Fractions D, E, and G were enriched in flavonoids, with Fr. E exhibiting the highest total flavonoid content (284.6 ± 5.2 mg QE/g), followed by Fr. G (190.0 ± 4.8 mg QE/g).

High condensed tannin contents were also observed in several fractions. The last-eluting fraction, Fr. G, exhibited the highest condensed tannin content (955.5 ± 22.0 mg CE/g), followed by Fr. F (900.8 ± 20.5 mg CE/g) and Fr. E (862.3 ± 17.9 mg CE/g). The condensed tannin contents of PE-MeOH and PE-H_2_O were 537.4 ± 10.1 and 425.4 ± 9.5 mg CE/g, respectively. These findings indicate that the phenolic-rich fractions of *P. equisetiforme*, particularly Fr. E–G, contain considerable levels of flavonoids and condensed tannins.

### 2.2. Antioxidant Activities

**Figure 1:**
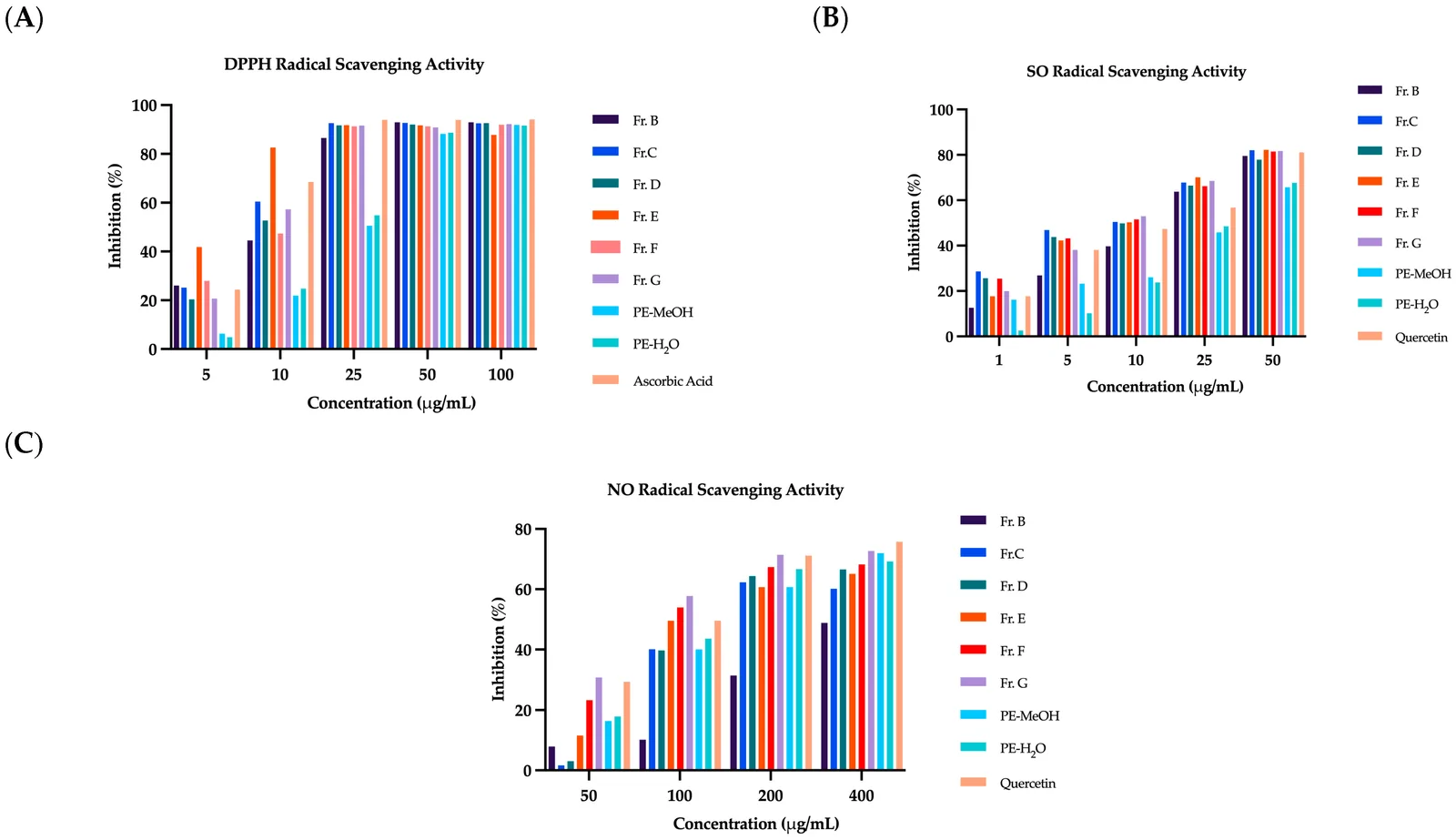
Radical-scavenging activities of *P. equisetiforme* extracts and fractions. (**A**) DPPH radical-scavenging activity of PE-MeOH, PE-H_2_O, and fractions Fr. B–G tested at 5–100 µg/mL, with ascorbic acid as the positive control. (**B**) Superoxide anion radical-scavenging activity of PE-MeOH, PE-H_2_O, and Fr. B–G tested at 1–100 µg/mL, with quercetin as the positive control. (**C**) Nitric oxide radical-scavenging activity of PE-MeOH, PE-H_2_O, and Fr. B–G tested at 50–400 µg/mL, with quercetin as the positive control. Results are expressed as percentage inhibition.

The DPPH radical-scavenging assay revealed that several fractions exhibited strong antioxidant potential. Fr. E showed the highest activity (IC_50_ = 6.00 µg/mL), followed closely by Fractions C, D, G, and F (IC_50_ = 8.52–10.88 µg/mL). These fractions showed scavenging capacities comparable to ascorbic acid (IC_50_ = 7.90 µg/mL), indicating the presence of potent hydrogen-donating phytochemicals. The crude extracts (PE–MeOH and PE–H_2_O) displayed weaker scavenging activity (IC_50_ > 22 µg/mL), suggesting that the active antioxidant constituents are highly enriched in specific subfractions rather than in the bulk extracts (Figure 1A).

The superoxide radical-scavenging assay showed clear differences in antioxidant potential among the fractions. The most active samples were Fractions G, F, C, and E (IC_50_ = 9.01–9.81 µg/mL), all of which showed stronger activity than quercetin (IC_50_ = 14.20 µg/mL). This indicates that *P. equisetiforme* contains highly potent antioxidant constituents enriched within these fractions. Fr. D (IC_50_ = 10.20 µg/mL) and Fr. B (IC_50_ = 16.42 µg/mL) showed moderate activity, while the crude extracts (PE–MeOH and PE–H_2_O) exhibited weaker effects (IC_50_ = 30.28 and 26.88 µg/mL, Figure 1B).

In the nitric oxide scavenging assay, Fr. G exhibited the strongest activity, showing the lowest IC_50_ value (85.61 µg/mL). This was followed by Fractions F (93.51 µg/mL) and E (103.69 µg/mL), the latter displaying potency comparable to that of the positive control quercetin (102.04 µg/mL). In contrast, Fractions C and D were less active (IC_50_ > 140 µg/mL), and Fraction B showed very low activity with an IC_50_ exceeding 400 µg/mL. Both crude extracts (PE–MeOH, IC_50_ ≈ 148 µg/mL; and PE–H_2_O, IC_50_ ≈ 128 µg/mL) also demonstrated moderate NO-scavenging efficiency, consistent with their limited dose–response increase (Figure 1C).

Both CUPRAC and FRAP assays revealed strong reducing capacity in the phenolic-rich fractions of *P. equisetiforme*. In the CUPRAC assay, Fr. D exhibited the highest electron-donating ability (940 mg GAE/g), greatly exceeding the crude extracts. The MeOH and water extracts showed lower activity (546.3 and 392.6 mg GAE/g, respectively), consistent with the dilution of active constituents in the unfractionated samples. In the FRAP assay, reducing power followed a similar pattern, with Fr. E showing the highest Fe^3+^-reducing capacity (826.9 mg TE/g). Other fractions displayed moderately high reducing potential, whereas the crude extracts yielded substantially lower FRAP values. The TEAC assay demonstrated uniformly high ABTS^+^· scavenging among the phenolic-enriched fractions. Fr. D showed the strongest response (474.4 mg Trolox/g), followed closely by Fractions F, E, and G (448–451 mg Trolox/g). Fractions B and C also exhibited considerable antioxidant capacity (443–447 mg Trolox/g). In contrast, the crude extracts showed reduced TEAC values (PE–MeOH: 340.4 mg/g; PE–H_2_O: 307.5 mg/g), reflecting their lower phenolic enrichment (Table 1).

### 2.3. Enzyme-Inhibition Activities

**Figure 2:**
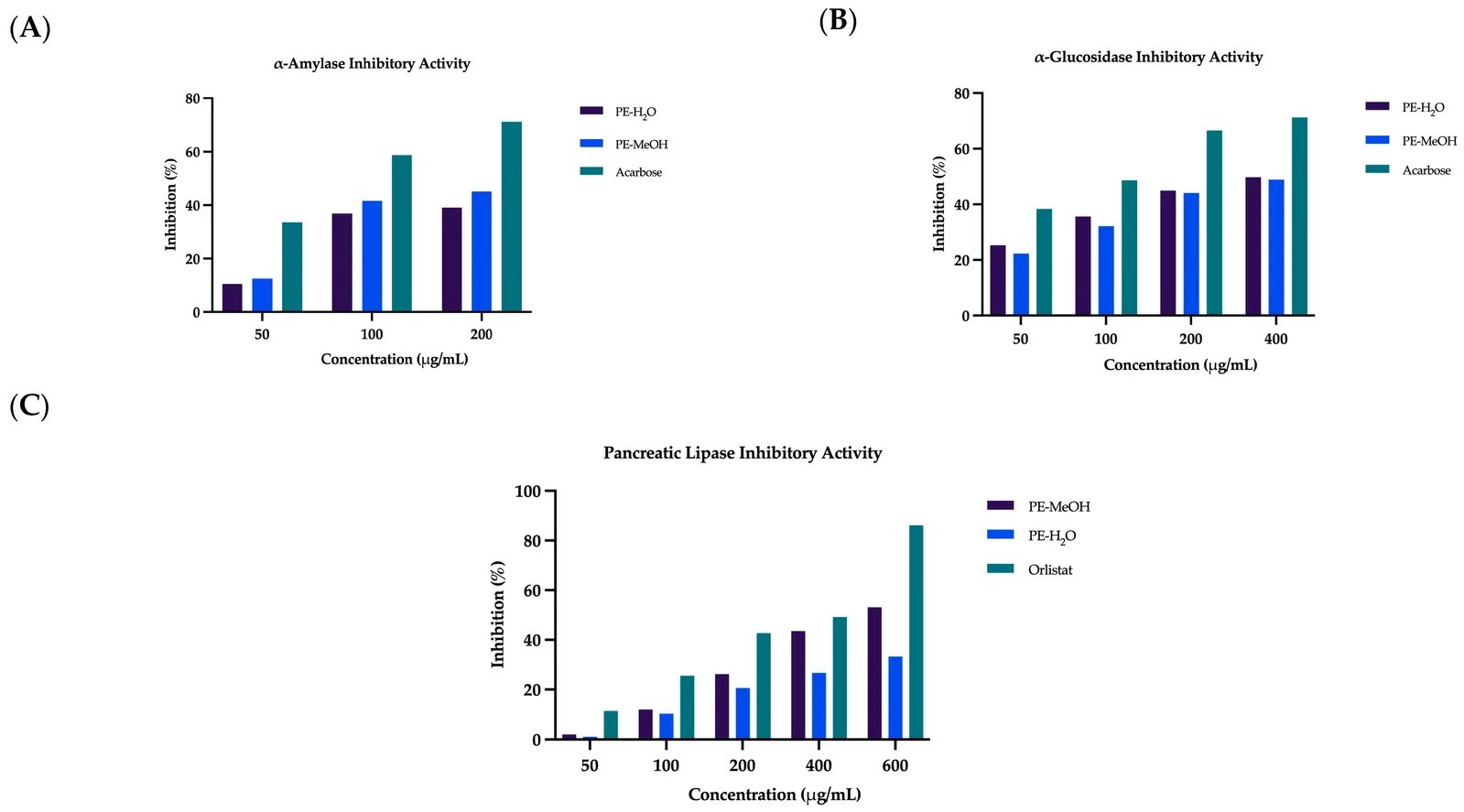
Enzyme-inhibitory activities of *P. equisetiforme* extracts. (**A**) α-amylase inhibitory activity of the methanolic extract (PE-MeOH) and aqueous extract (PE-H_2_O) tested at concentrations of 50–200 µg/mL. (**B**) α-glucosidase inhibitory activity of PE-MeOH and PE-H_2_O at concentrations of 50–400 µg/mL. (**C**) Pancreatic lipase inhibitory activity of PE-MeOH and PE-H_2_O at concentrations of 50–600 µg/mL. Results are expressed as percentage inhibition.

The methanolic (PE-MeOH) and defatted aqueous (PE-H_2_O) extracts of *P. equisetiforme* inhibited α-glucosidase activity in a dose-dependent manner and exhibited comparable inhibitory profiles, with IC_50_ values of 374.04 ± 12.42 µg/mL and 362.82 ± 10.91 µg/mL, respectively. In contrast, the α-amylase inhibition assay showed a stronger inhibitory effect for the aqueous extract, with a lower IC_50_ value (285.0 ± 8.62 µg/mL) compared to the methanolic extract (310.95 ± 9.85 µg/mL) (Table 2, Figure 2A,B).

The extracts were tested for their ability to inhibit pancreatic lipase, an approach relevant to controlling dietary fat absorption and obesity. The results are shown in Table 2. Orlistat, used as the positive control, showed an IC_50_ of 328.3 ± 11.1 μg/mL. PE-MeOH and PE-H_2_O inhibited pancreatic lipase with IC_50_ values of 519.2 ± 20.51 μg/mL and 868.5 ± 30.10 μg/mL, respectively (Table 2, Figure 2C).

### 2.4. Antioxidant and Enzyme-Inhibitory Activities of Catechin and Quercetin

Catechin and quercetin were selected as representative isolated phenolic compounds and evaluated individually in the DPPH and ABTS radical-scavenging assays and the α-glucosidase and pancreatic lipase inhibition assays. Catechin and quercetin exhibited concentration-dependent radical-scavenging activity. In the DPPH assay, catechin and quercetin showed IC_50_ values of 58.73 and 5.70 µg/mL, respectively. In the ABTS assay, the corresponding IC_50_ values were 42.58 and 3.91 µg/mL, respectively. Quercetin was more active than catechin in both assays. In the α-glucosidase assay, both compounds produced more than 50% inhibition at the lowest tested concentrations; therefore, their IC_50_ values were reported as <25 µg/mL for catechin and <12.5 µg/mL for quercetin. In the pancreatic lipase assay, catechin and quercetin showed IC_50_ values of 56.35 ± 1.26 and 18.62 ± 0.20 µg/mL, respectively (Table 3).

### 2.5. Glucose Uptake in 3T3-L1 Preadipocytes

**Figure 3:**
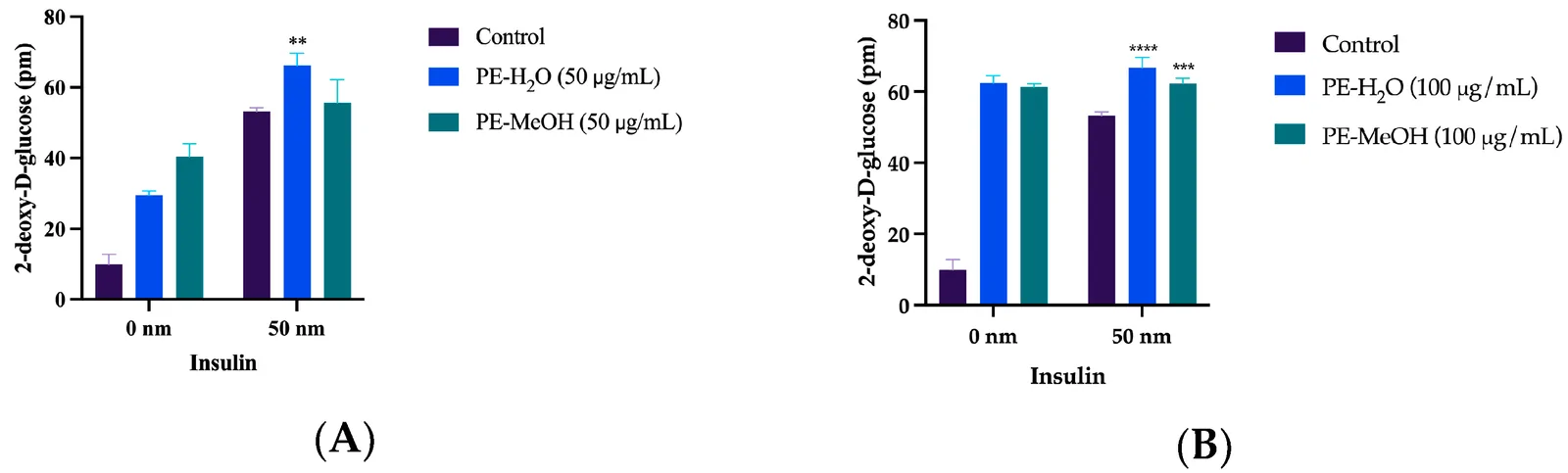
Effects of *P. equisetiforme* extracts on 2-deoxy-D-glucose uptake in 3T3-L1 preadipocytes in the absence or presence of insulin (50 nM). (**A**) PE-H_2_O and PE-MeOH tested at 50 µg/mL. (**B**) PE-H_2_O and PE-MeOH tested at 100 µg/mL. Data are presented as mean ± SD from three independent experiments (*n* = 3). Statistical analysis was performed separately for each extract concentration using ordinary two-way ANOVA followed by Šídák’s multiple-comparisons test. ** *p* < 0.01, *** *p* < 0.001, and **** *p* < 0.0001.

To evaluate the effects of *P. equisetiforme* extracts on cellular glucose uptake, 2-deoxy-D-glucose uptake was measured in 3T3-L1 preadipocytes in the absence and presence of insulin (50 nM). The results are shown in Figure 3A (50 μg/mL sample concentration) and Figure 3B (100 μg/mL). At 50 μg/mL (without insulin), both PE-H_2_O and PE-MeOH significantly increased glucose uptake compared with the untreated control (both *p* < 0.0001). In the presence of insulin, the PE-H_2_O–insulin combination produced significantly greater glucose uptake than insulin alone (*p* = 0.0017), whereas the PE-MeOH–insulin combination did not differ significantly from insulin alone (*p* = 0.6560). Two-way ANOVA revealed a significant insulin × treatment interaction at 50 µg/mL [F(2,12) = 24.65, *p* < 0.0001]. At 100 μg/mL in the absence of insulin, both PE-H_2_O and PE-MeOH significantly increased glucose uptake compared with the untreated control (both *p* < 0.0001). In the presence of insulin, glucose uptake was significantly greater with both PE-H_2_O plus insulin (*p* < 0.0001) and PE-MeOH plus insulin (*p* = 0.0003) than with insulin alone. A significant insulin × treatment interaction was also detected at 100 µg/mL [F(2,12) = 199.73, *p* < 0.0001].

### 2.6. Identification of Isolated Compounds

The isolated samples were characterized by combined analysis of 1D and 2D NMR experiments (^1^H NMR, ^13^C NMR, COSY, HSQC/HMQC, and HMBC) recorded on a Bruker Avance III spectrometer (Bruker, Rheinstetten, Germany), together withthe available mass-spectrometric data. PEC-2 and PEE-4 were obtained as inseparable mixtures, each containing two compounds. The assignments were established by interpretation of the spectroscopic data and comparison with previously reported values. The compound codes, structures, names, and molecular formulas are summarized in Table 4, while the spectroscopic data are presented below and the corresponding spectra are provided in the Supplementary Materials.

**PEC-2.** PEC-2 was obtained as a mixture of 3-*O*-acetyl-(−)-epicatechin (PEC-2a) and liquiritin (PEC-2b).

**3-*O*-Acetyl-(−)-epicatechin (PEC-2a).** Molecular formula: C_17_H_16_O_7_. Positive-ion full-scan ESI-MS *m*/*z* 333 [M + H]^+^ (calcd for C_17_H_17_O_7_^+^, 333.0969). ^1^H NMR (DMSO-d_6_) δ_H_ 6.79, 6.67, and 6.63 (B-ring aromatic protons), 5.93 and 5.77 (H-6 and H-8), 4.90–4.97 (H-2), 5.15 (H-3), 2.65–2.90 (H_2_-4), and 1.92 (s, 3H, OCOCH_3_); ^13^C NMR (DMSO-d_6_) δ_C_ 170.13 (OCOCH_3_), 129.70, 116.36, 115.59, 100.73, 95.94, 94.65, 77.49, 69.20 (C-3), and 21.34 (OCOCH_3_). The HSQC spectrum showed the diagnostic H-3/C-3 correlation at δ_H_/δ_C_ 5.15/69.20. In the HMBC spectrum, the acetyl methyl protons at δ_H_ 1.93 correlated with the ester carbonyl carbon at δ_C_ 170.13. The acetyl resonances and the downfield position of H-3 supported the assignment of the acetyl substituent at C-3. These data were consistent with those reported for 3-*O*-acetyl-(−)-epicatechin [25] (Figures S1–S5).

**Liquiritin (PEC-2b).** Molecular formula: C_21_H_22_O_9_. Positive-ion full-scan ESI-MS *m*/*z* 419 [M + H]^+^ (calcd for C_21_H_23_O_9_^+^, 419.1337). ^1^H NMR (DMSO-d_6_) δ_H_ 7.64 and 6.84 (B-ring aromatic protons), 6.51 and 6.34 (H-6 and H-8), 5.52 (H-2), 4.97 (H-1″), 3.15 (H-3a), 2.65 (H-3b), and 3.20–3.73 (glucosyl protons); ^13^C NMR (DMSO-d_6_) δ_C approximately 190.4 (C-4), 163.6, 158.0, 129.7, 128.9, 116.6, 103.1, 100.3 (C-1″), 79.1, 77.5, 76.4, 73.7, 70.2, and 60.7 (C-6″). The HMBC correlations from H-3a/H-3b to the C-4 carbonyl supported the flavanone skeleton. The β-glucopyranosyl unit was indicated by the anomeric signal at δ_H_ /δ_C 4.97/approximately 100.3. The assignment was consistent with the reported spectroscopic data for liquiritin [26] (Figures S1–S5).

**Catechin (PEE-1).** Molecular formula: C_15_H_14_O_6_. Positive-ion full-scan ESI-MS *m*/*z* 313 [M + Na]^+^ (calcd for C_15_H_14_NaO_6_^+^, 313.0683) ^1^H NMR (300 MHz, CD_3_OD) δ_H_ 6.84 (d, J = 2.0 Hz, H-2′), 6.76 (d, J = 8.1 Hz, H-5′), 6.72 (dd, J = 8.3, 2.0 Hz, H-6′), 5.93 (d, J = 2.2 Hz, H-8), 5.86 (d, J = 2.2 Hz, H-6), 4.56 (d, J = 7.3 Hz, H-2), 3.97 (m, H-3), 2.85 (dd, J = 16.0, 5.4 Hz, H-4a), and 2.50 (dd, J = 16.0, 8.0 Hz, H-4b); ^13^C NMR (75 MHz, CD_3_OD) δ_C_ 156.8 (C-5/C-7/C-9), 146.4 (C-3′ and C-4′), 132.4 (C-1′), 120.2 (C-6′), 116.1 (C-5′), 115.3 (C-2′), 101.0 (C-10), 96.3 (C-8), 95.7 (C-6), 83.0 (C-2), 69.0 (C-3), and 28.7 (C-4). The COSY spectrum showed the sequential correlations H-2/H-3 and H-3/H-4a/H-4b. The HSQC spectrum confirmed the diagnostic correlations at δ_H_ / δ_C_ 4.55/82.99 (H-2/C-2), 3.96/68.97 (H-3/C-3), 5.93/96.29 (H-8/C-8), 5.85/95.59 (H-6/C-6), 6.84/115.38 (H-2′/C-2′), and 6.75/116.15 (H-5′/C-5′). The relatively large coupling constant between H-2 and H-3 was consistent with the trans relative configuration of catechin [27] (Figures S6–S9).

**PEE-2.** PEE-2 was identified as tamarixetin based on its diagnostic NMR and mass-spectrometric data. Molecular formula: C_16_H_12_O_7_. Positive-ion full-scan ESI-MS *m*/*z* 317 [M + H]^+^ (calcd for C_16_H_13_O_7_^+^, 317.0656). ^1^H NMR (300 MHz, CD_3_OD) δ_H_ 7.76–7.73 (m, 2H, H-2′ and H-6′), 7.06 (d, *J* = 8.6 Hz, H-5′), 6.40 (d, *J* = 2.0 Hz, H-8), 6.19 (d, *J* = 2.0 Hz, H-6), and 3.93 (s, 3H, 4′-OCH_3_). The corresponding carbon assignments were δ_C_ 177.6 (C-4), 165.8 (C-7), 162.7 (C-5), 158.4 (C-9), 150.8 (C-4′), 148.2 (C-2), 147.7 (C-3′), 137.2 (C-3), 125.6 (C-1′), 121.6 (C-6′), 115.8 (C-2′), 112.4 (C-5′), 104.7 (C-10), 99.4 (C-6), 94.5 (C-8), and 56.5 (4′-OCH_3_). The HSQC spectrum showed the diagnostic δ_H_ / δ_C_ correlations 7.75/121.63 (H-6′/C-6′), 7.74/115.75 (H-2′/C-2′), 7.07/112.38 (H-5′/C-5′), 6.40/94.50 (H-8/C-8), 6.19/99.40 (H-6/C-6), and 3.94/56.45 (4′-OCH_3_). The H-5′/H-6′ COSY correlation supported the 1,2,4-trisubstituted B-ring system, while the HMBC correlation from 4′-OCH_3_ to C-4′ supported the location of the methoxy group. These data were consistent with the reported spectroscopic data for tamarixetin [28] (Figures S10–S14).

**PEE-3.** PEE-3 was identified as quercetin based on its characteristic NMR data and comparisons with published values. Molecular formula: C_15_H_10_O_7_. Negative-ion full-scan ESI-MS *m*/*z* 301 [M − H]^−^ (calcd for C_15_H_9_O_7_^−^, 301.0354). The ^1^H NMR spectrum (300 MHz, DMSO-d_6_) showed signals at δ_H_ 12.48 (s, 5-OH), 7.67 (d, J ≈ 2.1 Hz, H-2′), 7.54 (dd, *J* ≈ 8.5, 2.1 Hz, H-6′), 6.89 (d, *J* ≈ 8.5 Hz, H-5′), 6.41 (d, *J* ≈ 2.0 Hz, H-8), and 6.19 (d, *J* ≈ 2.0 Hz, H-6). The corresponding ^13^C NMR/APT data (75 MHz, DMSO-d_6_) were δ_C_ 176.0 (C-4), 164.0 (C-7), 160.8 (C-5), 156.3 (C-9), 147.9 (C-4′), 146.7 (C-2), 145.2 (C-3′), 135.9 (C-3), 122.2 (C-1′), 120.2 (C-6′), 115.7 (C-5′), 115.3 (C-2′), 103.1 (C-10), 98.3 (C-6), and 93.5 (C-8). The H-5′/H-6′ COSY correlation further supported the 1,2,4-trisubstituted B-ring system. These data were consistent with quercetin and published NMR data [29] (Figures S15–S17).

**PEE-4** was obtained as an inseparable mixture of quercetin-3-*O*-glucoside (isoquercitrin, PEE-4a) and quercetin-3-*O*-galactoside (hyperoside, PEE-4b). Both constituents have the molecular formula C_21_H_20_O_12_. Their assignments were based primarily on diagnostic 1D-2D NMR data together with published spectroscopic data.

PEE-4a (isoquercitrin): ^1^H NMR (300 MHz, DMSO-d_6_) δ_H_ 7.71 (d, *J* = 2.2 Hz, H-2′), 7.59 (dd, *J* = 8.5, 2.2 Hz, H-6′), 6.87 (d, *J* = 8.4 Hz, H-5′), 6.40 (d, *J* = 2.1 Hz, H-8), 6.21 (d, *J* = 2.1 Hz, H-6), 5.26 (d, *J* = 7.6 Hz, H-1″), 3.71 (dd, *J* = 11.9, 2.3 Hz, H-6″a), 3.59 (overlapped, H-6″b), 3.47 (overlapped, H-2″), 3.42 (t, *J* = 8.9 Hz, H-3″), 3.35 (t, *J* = 9.1 Hz, H-4″), and 3.22 (ddd, *J* = 9.6, 5.1, 2.4 Hz, H-5″); ^13^C NMR (assigned from HSQC/HMBC, DMSO-d_6_) δ_C_ 179.7 (C-4), 166.2 (C-7), 163.2 (C-5), 159.0 (C-2), 158.9 (C-9), 150.1 (C-4′), 146.0 (C-3′), 135.9 (C-3), 123.3 (C-6′), 123.1 (C-1′), 117.7 (C-2′), 116.1 (C-5′), 105.9 (C-10), 104.4 (C-1″), 100.0 (C-6), 94.9 (C-8), 78.6 (C-5″), 78.3 (C-3″), 75.9 (C-2″), 71.4 (C-4″), and 62.7 (C-6″). The HMBC correlation H-1″ (δ_H_ 5.26) → C-3 (δ_C_ 135.9) supported 3-*O*-glycosylation [29] (Figures S18–S22).

PEE-4b (hyperoside): ^1^H NMR (300 MHz, DMSO-d_6_) δ_H_ 7.84 (d, *J* = 2.2 Hz, H-2′), 7.59 (dd, *J* = 8.5, 2.2 Hz, H-6′), 6.87 (d, *J* = 8.5 Hz, H-5′), 6.41 (d, *J* = 2.1 Hz, H-8), 6.21 (d, *J* = 2.1 Hz, H-6), 5.17 (d, *J* = 7.7 Hz, H-1″), 3.85 (br d, *J* ≈ 3.0 Hz, H-4″), 3.82 (dd, *J* = 9.6, 7.8 Hz, H-2″), 3.64 (dd, *J* = 11.2, 6.0 Hz, H-6″a), 3.57 (overlapped, H-6″b), 3.56 (overlapped, H-3″), and 3.47 (overlapped, H-5″); ^13^C NMR (assigned from HSQC/HMBC, DMSO-d_6_) δ_C_ 179.7 (C-4), 166.2 (C-7), 163.2 (C-5), 159.0 (C-2), 158.6 (C-9), 150.1 (C-4′), 146.0 (C-3′), 135.8 (C-3), 123.1 (C-6′), 123.0 (C-1′), 117.9 (C-2′), 116.2 (C-5′), 105.8 (C-10), 105.5 (C-1″), 100.0 (C-6), 94.9 (C-8), 77.4 (C-5″), 75.3 (C-3″), 73.3 (C-2″), 70.2 (C-4″), and 62.1 (C-6″). The HMBC correlation H-1″ (δ_H_ 5.17) → C-3 (δ_C_ 135.8) supported 3-*O*-glycosylation. The relatively small H-3″/H-4″ coupling was consistent with a galactopyranosyl residue [29] (Figures S18–S22).

## 3. Discussion

The results confirm that *P. equisetiforme* is very rich in polyphenolic compounds and possesses strong antioxidant properties, as well as moderate inhibitory effects on carbohydrate- and lipid-digesting enzymes and an ability to enhance glucose uptake in cells.

The potent antioxidant activities of *P. equisetiforme* extracts observed in our assays are consistent with previous studies. The total phenolic content (e.g., 523 mg GAE/g for the methanol extract and up to 835 mg GAE/g for Fr. E) is well above the range reported (31–113 mg GAE/g dw for various populations) [7]. In the DPPH radical-scavenging assay, Fraction E showed the strongest activity (IC_50_ = 6.00 µg/mL), followed by Fractions C, D, G, and F (IC_50_ = 8.52–10.88 µg/mL). Fraction E was slightly more active than the ascorbic acid reference (IC_50_ = 7.90 µg/mL), whereas the crude extracts were less active (IC_50_ > 22 µg/mL). These results indicate that fractionation concentrated the antioxidant constituents in specific fractions. The superoxide and nitric oxide scavenging activities were also notable. In the superoxide anion radical-scavenging assay, Fractions G, F, C, and E were the most active samples, with IC_50_ values ranging from 9.01 to 9.81 µg/mL. Under the assay conditions, these fractions showed greater activity than the quercetin reference (IC_50_ = 14.20 µg/mL). Fraction D also showed pronounced activity (IC_50_ = 10.20 µg/mL), whereas Fraction B and the crude extracts were less active. In the nitric oxide radical-scavenging assay, Fraction G was the most active fraction (IC_50_ = 85.61 µg/mL), followed by Fractions F (93.51 µg/mL) and E (103.69 µg/mL). Fraction E showed activity comparable to the quercetin reference (IC_50_ = 102.04 µg/mL), whereas Fractions C and D and the crude extracts were less active. Fraction B did not reach 50% inhibition within the tested concentration range.

In the FRAP and CUPRAC assays, *P. equisetiforme* fractions showed substantial reducing capacity. Fr. E showed the highest FRAP value (826.9 mg TE/g), whereas the CUPRAC value of Fr. D reached 940 mg TE/g. These findings may reflect the combined contribution of multiple phenolic constituents, including tannins. Strong FRAP activity has also been reported for green tea flavan-3-ols and oligomeric proanthocyanidins, with antioxidant capacity correlating with total polyphenol content [30]. Similarly, isolated constituents of *Polygonum aviculare* have demonstrated antioxidant and α-glucosidase-inhibitory activities, while extracts of four *Polygonum* species exhibited varying but notable antioxidant and reducing capacities [31,32]. Consistently, ethanol and hydroethanolic extracts of *P. equisetiforme* exhibited inhibitory effects on α-amylase and α-glucosidase, along with strong antioxidant capacity, further supporting the metabolic relevance of this species in the context of diabetes and obesity [13].

Pancreatic lipase inhibition by *P. equisetiforme* is a novel finding. The methanol extract showed an IC_50_ of 519.2 μg/mL, while the water extract had a weaker effect (IC_50_ = 868.5 μg/mL), compared to the standard orlistat (IC_50_ = 328.3 μg/mL). This indicates mild-to-moderate pancreatic lipase inhibition under the present in vitro conditions.

Effects on cellular glucose uptake: *P. equisetiforme* extracts increased 2-deoxy-D-glucose uptake in 3T3-L1 preadipocytes, particularly at 100 µg/mL. Quercetin and catechin have previously been reported to modulate insulin-signaling pathways or GLUT4 translocation, suggesting that phenolic constituents may contribute to the observed increase in glucose uptake [33,34,35]. However, these findings indicate enhanced cellular glucose uptake rather than directly demonstrating insulin-mimetic or insulin-sensitizing activity. Further studies are warranted to clarify the underlying molecular mechanisms [17,33,36].

Based on the bioactivity observed in the active fractions of *P. equisetiforme*, the isolated compounds may contribute to the overall biological activity of the plant.

The acetylated flavanol epicatechin-3-acetate (PEC-2a), although structurally derived from the widely distributed epicatechin, is reported here for the first time in the *Polygonum* genus. While epicatechin-3-acetate has previously been described mainly through synthetic or semi-synthetic approaches, its natural occurrence has been only rarely documented, increasing the phytochemical relevance of its isolation from *P. equisetiforme*. Epicatechin and related flavan-3-ols have been shown to inhibit key digestive enzymes involved in metabolic disorders. In particular, extracts and components of black tea containing epicatechin derivatives significantly inhibited α-amylase, α-glucosidase, and pancreatic lipase in vitro [37]. Moreover, epicatechin-3-*O*-acetate isolated from Rhizophora stylosa exhibited strong DPPH radical-scavenging activity (IC_50_ = 15.3 μg/mL) [25].

Liquiritin (PEC-2b), a flavanone glycoside (liquiritigenin-4′-*O*-β-D-glucopyranoside), has previously been identified in *Polygonum aviculare* [38]. Liquiritin exhibits α-glucosidase inhibitory activity, with the aglycone liquiritigenin being more potent than its glycosylated form [39]. Similarly, (+)-catechin (PEE-1), a widely distributed flavanol, has well-established antioxidant properties and has been reported to inhibit α-glucosidase. More broadly, catechins have demonstrated variable effects on α-amylase, pancreatic lipase, and glucose transport depending on their structures and the experimental models used [34,40,41,42]. The ubiquitous flavonol quercetin (PEE-3), well-documented in the *Polygonum* genus, shows strong inhibitory effects on α-glucosidase, α-amylase, and pancreatic lipase [1,43]. Its methylated derivative tamarixetin (PEE-2), reported here as an aglycone for the first time in *P. equisetiforme*, was previously isolated from *P. hydropiper* in the form of tamarixetin 3-glucoside-7-sulfate [44]. The glycosides quercetin-3-*O*-glucoside (PEE-4a, isoquercitrin) and quercetin-3-*O*-galactoside (PEE-4b, hyperoside), both present in various *Polygonum* species, retain significant antioxidant properties and inhibit α-glucosidase and α-amylase to a moderate degree [44,45,46].

In the present study, direct evaluation of catechin and quercetin indicated that both compounds may contribute to the antioxidant and enzyme-inhibitory activities of *P. equisetiforme*. Quercetin was more active than catechin in the DPPH and ABTS assays and exhibited stronger pancreatic lipase inhibition. Both compounds produced more than 50% α-glucosidase inhibition at the lowest concentration tested. Overall, the findings demonstrate that the activities of individual constituents vary according to the experimental target and do not support attributing the activity of the extracts and fractions to a single compound. Since the remaining isolated constituents were not evaluated individually, their specific contributions require further investigation.

Consistent with these reports, the bioactivity profile of *P. equisetiforme* indicates that its phenolic constituents contribute to metabolic enzyme inhibition and glucose-uptake enhancement, alongside high antioxidant capacity, with these in vitro activities potentially relevant to metabolic processes associated with obesity and diabetes.

## 4. Materials and Methods

### 4.1. Plant Material

Aerial parts of *P. equisetiforme* were collected from Antalya, Türkiye, in 2013. The plant specimen was identified by Prof. Dr. Hayri Duman and deposited at the Herbarium of Hacettepe University Faculty of Pharmacy (HUEF 13033, 13034).

### 4.2. Extraction

The aerial parts of the plant were dried in a shaded, moisture-free environment. After complete drying, the plant material was powdered using a laboratory grinder. A total of 400 g of powdered *P. equisetiforme* was macerated three times with 2 L of methanol at 40 °C in a water bath. The combined extracts were filtered and concentrated under reduced pressure using a rotary evaporator to obtain 69.83 g of a dense extract (yield: 17.45%, PE-MeOH). The crude extract was dissolved in 250 mL of water and shaken five times with 100 mL of n-hexane (5 × 100 mL) to remove chlorophylls and lipophilic compounds. The remaining aqueous phase was evaporated to dryness, yielding 55.90 g of dried extract (yield: 13.9%, PE-H_2_O).

### 4.3. Phytochemical Studies

#### 4.3.1. Preliminary Fractionation

The defatted aqueous extract (40 g) of *P. equisetiforme* (PE-H_2_O) was subjected to polyamide (Sigma-Aldrich, St. Louis, MO, USA), column chromatography and eluted with a gradient of increasing methanol concentration in water. The gradient elution was as follows: 100% water (fractions 1–4), 20% MeOH (fractions 5–8), 40% MeOH (fractions 9–12), 60% MeOH (fractions 13–15), 80% MeOH (fractions 16–18), and 100% MeOH (fractions 19–28). Fractions were collected in approximately 80–400 mL portions for each step. A total of 28 initial fractions were obtained, which were then combined based on thin-layer chromatography (TLC) analysis performed using precoated silica gel 60 F_254_ aluminum plates (Merck, Darmstadt, Germany). Fractions with similar TLC profiles were combined, resulting in seven main fractions, labeled as Fr. 1–4 (Fr. A, 17.99 g), Fr. 5–8 (Fr. B, 3.56 g), Fr. 9–12 (Fr. C, 2.39 g), Fr. 13–15 (Fr. D, 1.52 g), Fr.1 6–18 (Fr. E, 1.54 g), Fr. 19–22 (Fr. F, 1.08 g), and Fr. 23–28 (Fr. G, 0.61 g).

#### 4.3.2. Purification of Compounds

Based on the preliminary phytochemical and antioxidant results, fractions C, D, and E were selected for further purification using different chromatographic techniques.

Fraction C (2.39 g) was subjected to silica gel (70–230 mesh; Merck, Darmstadt, Germany) column chromatography using an ethyl acetate–methanol–water gradient (100:5:1 → 100% MeOH). Fraction 9 was subsequently purified by preparative TLC using the same solvent system, yielding PEC-2 (3.3 mg). PEC-2 was obtained as a two-component mixture whose constituents were assigned as 3-*O*-acetyl-(−)-epicatechin (PEC-2a) and liquiritin (PEC-2b) based on combined evaluation of the available 1D and 2D NMR and supporting mass-spectrometric data.

Fraction D (50 mg) was subjected to preparative TLC using chloroform–methanol–water (61:32:7, *v*/*v*/*v*) as the mobile phase, yielding PED-1 (14.4 mg). Based on its diagnostic NMR profile, PED-1 was conservatively described as a catechin-containing isolate and was not counted as an additional structurally characterized compound.

Fraction E (1.54 g) was initially subjected to polyamide column chromatography using a stepwise H_2_O–MeOH gradient (90:10 → 100% MeOH). Selected fractions were further purified by reversed-phase (Merck, Darmstadt, Germany) column chromatography using MeOH–H_2_O (5:95 → 50:50), yielding catechin (PEE-1, 20.2 mg) and tamarixetin (PEE-2, 10.7 mg). Fractions 53–58 were purified by polyamide column chromatography using MeOH–H_2_O (10:90 → 100:0), yielding quercetin (PEE-3, 38.3 mg). Fractions 59–65 were subjected to reversed-phase column chromatography using MeOH–H_2_O (10:90 → 50:50), yielding PEE-4 (25.0 mg), which contained isoquercitrin (PEE-4a) and hyperoside (PEE-4b).

**Figure 4:**
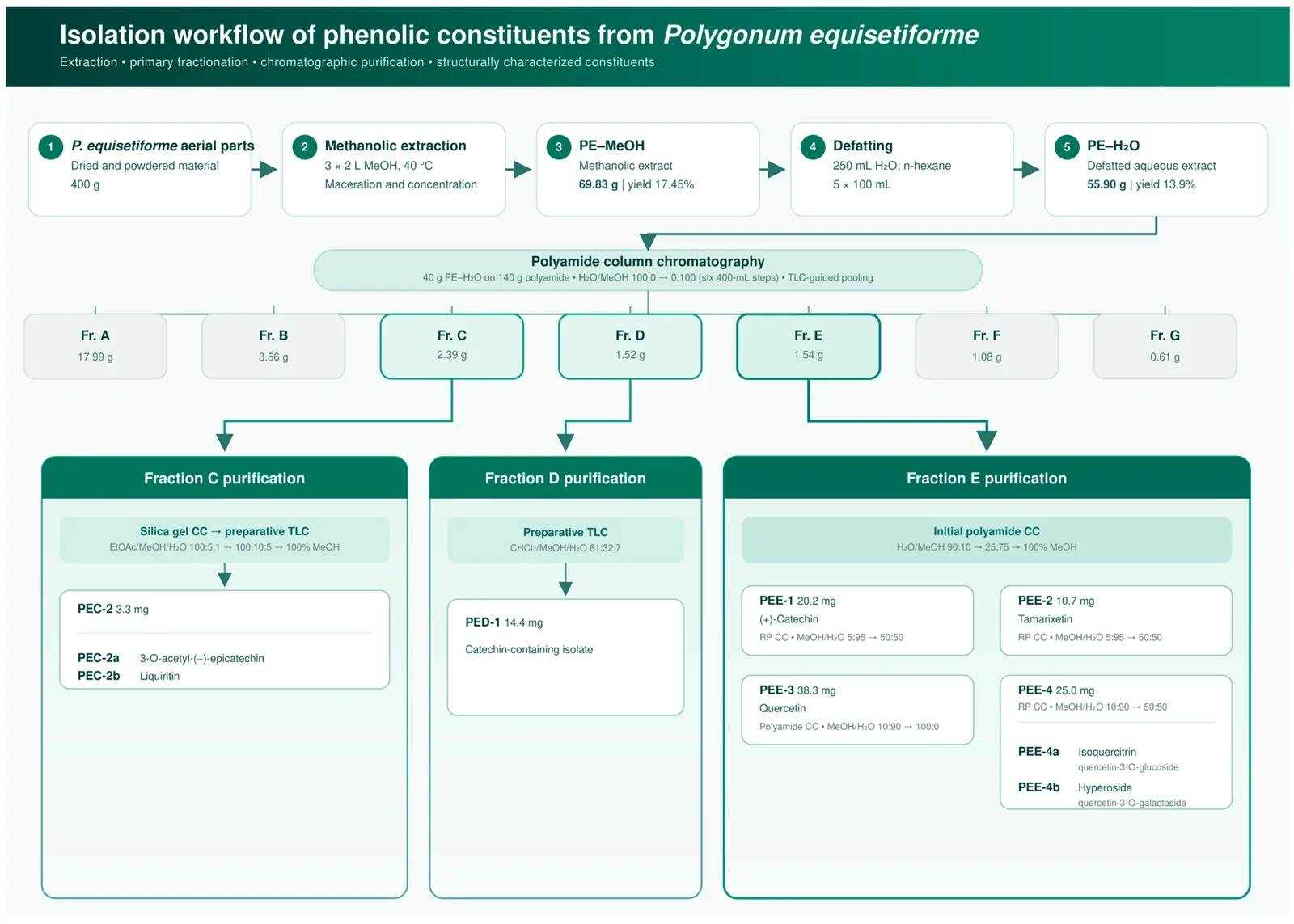
Extraction, fractionation, and isolation workflow for the phenolic constituents of *P. equisetiforme*.

The extraction, fractionation, purification, and compound-identification workflow is summarized in Figure 4.

#### 4.3.3. Phytochemical Content Assays

Folin–Ciocalteu reagent, gallic acid, aluminum chloride, potassium acetate, vanillin, and (+)-catechin were purchased from Sigma-Aldrich (St. Louis, MO, USA). Absorbance measurements were performed using a µQuant microplate spectrophotometer (BioTek Instruments, Winooski, VT, USA).

The total contents of key secondary metabolite classes (phenolics, flavonoids, and tannins) in the extracts and fractions were quantified by colorimetric assays:

Total Phenolic Content (TPC): Total phenolics were determined by the Folin–Ciocalteu colorimetric method with slight modification [47,48]. Briefly, each sample (extract or fraction) solution was mixed with diluted (1:10) Folin–Ciocalteu’s reagent. The mixture was shaken, and sodium carbonate (Na_2_CO_3_) solution (7.5%) was added. After 2 h of incubation, absorbance was measured at 765 nm. Gallic acid was used as a reference for calibration (0–400 μg/mL), and results were expressed as milligrams of gallic acid equivalents per gram of sample (mg GAE/g sample).

Total Flavonoid Content (TFC): Total flavonoids were measured by the aluminum chloride (AlCl_3_) colorimetric assay with slight modification [48,49]. In total, 25 μL of sample solutions were mixed with 75 μL of 95% ethanol, 5 μL of 10% aluminum chloride (AlCl3), 5 μL of 1 M potassium acetate (KCH_3_COO), and 140 μL of deionized (DI) water. After 30 min of incubation at ambient temperature, absorbance was read at 415 nm. Quercetin was used as a reference for calibration (0–400 μg/mL). TFC was expressed as milligrams of quercetin equivalents per gram of sample (mg QE/g sample).

Total Condensed Tannins (Proanthocyanidins) Content: The vanillin–HCl colorimetric assay was employed to estimate condensed tannins [50]. In this assay, sample solutions were reacted with vanillin in 4% methanol and concentrated hydrochloric acid (HCl). After 15 min of incubation at ambient temperature, absorbance was measured at 500 nm. (+)-Catechin was used as a reference, and tannin content was expressed as mg catechin equivalents per g of sample (mg CE/g sample).

Each assay was performed in triplicate for accuracy. Appropriate blanks (reagents without sample) were included, and calibration curves for standards were generated (all R^2^ > 0.99). The data were reported as mean ± standard deviation (SD).

Antioxidant Activity Assays

DPPH, ABTS, nitroblue tetrazolium (NBT), sodium nitroprusside, neocuproine, TPTZ, Trolox, quercetin, and ascorbic acid were purchased from Sigma-Aldrich (St. Louis, MO, USA). Absorbance measurements were performed using a µQuant microplate spectrophotometer (BioTek Instruments, Winooski, VT, USA).

A series of in vitro antioxidant assays were applied to *P. equisetiforme* extracts and fractions. All measurements were done in triplicate.

DPPH Radical-Scavenging Assay

DPPH radical-scavenging capacity was determined by the method described with slight modification [48,51]. Various concentrations of each sample (1–100 μg/mL) were mixed with 1 mM DPPH solution in methanol. After 30 min of incubation in the dark, the decrease in absorbance was recorded at 517 nm. The inhibition percentage of DPPH radical was calculated using the following formula:

Inhibition % = [(A_blank_ − A_sample_)/A_blank_] × 100,

where A_blank_ is the absorbance of the blank (containing methanol instead of sample) and A_sample_ is the absorbance of the extracts or fractions. Ascorbic acid was used as a positive control. The concentration of sample required to scavenge 50% of DPPH (IC_50_) was determined from the plotted graph of scavenging activity against the concentrations of the sample.

Superoxide Anion Radical (O_2_•^−^)-Scavenging Assay

Superoxide scavenging was measured by the alkaline DMSO/nitroblue tetrazolium (NBT) method with slight modification [52,53]. Briefly, 30 µL of sample solutions prepared in DMSO (1–50 µg/mL) and 10 µL of NBT solution were added to each well of a 96-well plate. Then, 100 µL of alkaline DMSO was added to each well and absorbances were measured at 560 nm. The percent inhibition of NBT reduction was calculated and IC_50_ values were derived from dose–response curves. Quercetin was used as a positive control.

Nitric Oxide (NO) Radical-Scavenging Assay

The NO radical-scavenging activity was determined by the method described with slight modification [53,54]. A total of 60 µL of samples (50–400 µg/mL) were incubated with 60 µL of 10 mM sodium nitroprusside in 50 mM phosphate buffer (pH 7.4) at 25 °C for 2 h. Afterwards, the reaction mixture was mixed with 120 µL of Griess reagent (1% sulfanilamide, 0.1% N-1-naphthylethylenediamine in 2% phosphoric acid) and left for 10 min at room temperature. Absorbance at 577 nm was measured, and % NO scavenging was calculated versus a buffer blank using the following formula:

Inhibition % = [(A_blank_ − A_sample_)/A_blank_] × 100.

Quercetin was used as a positive control.

Cupric Ion Reducing Antioxidant Capacity (CUPRAC) Assay

The CUPRAC assay was performed according to the method described with slight modification [48,55]. Equal volumes (50 µL) of copper (II) chloride (CuCl_2_) (0.01 M), neocuproine (7.5 mM) and ammonium acetate (NH_4_OAc) buffer (pH 7.0; 1 M) were mixed with 25 µL of sample solutions and DI water. After 30 min standing at ambient temperature, absorbance was measured at 450 nm. Gallic acid was used as a reference for calibration. Antioxidant capacity was expressed as mg GAE/g sample.

Ferric Reducing Antioxidant Power (FRAP) Assay

Fresh FRAP reagent (300 mM acetate buffer pH 3.6:10 mM TPTZ in 40 mM HCl:20 mM FeCl_3_·6H_2_O = 10:1:1) was mixed with sample (10–100 µg/mL) or Trolox standard (0–500 µM) and incubated for 10 min at 37 °C. Absorbance at 593 nm was recorded, and results are expressed as mg TE/g sample (mean ± SD, *n* = 3) [56,57].

Trolox Equivalent Antioxidant Capacity (TEAC) Assay

ABTS radical cation scavenging activity was determined by the method described with slight modification [48,58]. ABTS•^+^ was generated by reacting 7 mM ABTS with 2.45 mM K_2_S_2_O_8_ in the dark for 16 h, then diluted to A_734_ = 0.70 ± 0.02. Next, 20 µL of sample was mixed with 200 µL of ABTS•^+^ and incubated for 6 min. Absorbance was measured at 734 nm. Trolox was used as a reference. Results were expressed in terms of Trolox equivalent antioxidant capacity (TEAC) (mg TE/g sample).

### 4.4. Biological Activity Studies

#### 4.4.1. Enzyme-Inhibition Assays

The inhibitory effects of extracts on key enzymes relevant to type 2 diabetes and obesity were evaluated. Yeast α-glucosidase, porcine pancreatic α-amylase, porcine pancreatic lipase, acarbose, and orlistat were purchased from Sigma-Aldrich (St. Louis, MO, USA).

α-Glucosidase Inhibition: Inhibition of α-glucosidase (a carbohydrate-hydrolyzing enzyme in the small intestine) was measured using yeast α-glucosidase and a chromogenic substrate, p-nitrophenyl-α-D-glucopyranoside (pNPG). The assay was carried out in 0.1 M phosphate buffer (pH 6.8) in 96-well plates. Sample solutions (at 50, 100, 200, 400 μg/mL final concentrations) were pre-incubated with α-glucosidase enzyme (0.1 U/mL) at 37 °C for 10 min. Then pNPG (5 mM) was added and the mixture was incubated for another 20 min. The reaction was stopped by adding 0.1 M Na_2_CO_3_. The amount of released p-nitrophenol was quantified by reading absorbance at 405 nm. Acarbose was used as a positive control. Percent inhibition was calculated relative to a solvent control (enzyme + substrate without inhibitor). IC_50_ values were determined from inhibition vs. concentration plots [53,59].

α-Amylase Inhibition: Inhibition of pancreatic α-amylase was tested by a similar approach. Porcine pancreatic α-amylase was incubated with samples (50–200 μg/mL) in phosphate buffer (pH 6.9) at 37 °C. Soluble starch (1%, *w*/*v*) was then added as substrate and incubated for 15 min. The reaction was stopped by adding 3,5-dinitrosalicylic acid (DNS) reagent and heating (90 °C, 5 min) to develop a colored product from reducing sugars. After cooling, the absorbance was measured at 540 nm. Acarbose was used as a positive control. Percentage inhibition at each concentration was calculated, and IC_50_ values were obtained [60,61].

Pancreatic Lipase Inhibition: Porcine pancreatic lipase was prepared in Tris-HCl buffer (pH 7.5). Samples (dissolved in DMSO, tested at 50–600 μg/mL) were pre-incubated with the enzyme at 37 °C for 5 min. Then, 0.1 mM p-nitrophenyl-laurate substrate (in Tris buffer with 0.1% Triton X-100) was added and the mixture was incubated at 37 °C for 30 min. Lipase activity releases p-nitrophenol, which was measured at 405 nm. Orlistat (a clinically used lipase inhibitor) was used as the positive control. The IC_50_ for lipase inhibition was determined from the decrease in absorbance relative to the control.

All enzyme assays were performed in triplicate. Results are reported as mean ± SD for percentage inhibition at high concentrations and IC_50_ values [62,63].

Catechin and quercetin were additionally evaluated as individual compounds using the same DPPH, ABTS, α-glucosidase, and pancreatic lipase assay procedures. In the DPPH and ABTS assays, catechin was tested at 3.125–100 µg/mL and quercetin at 6.25–200 µg/mL. In the α-glucosidase assay, catechin and quercetin were tested at 25–400 and 12.5–200 µg/mL, respectively. Both compounds were tested at 12.5–200 µg/mL in the pancreatic lipase assay. For these compounds, DPPH and ABTS activities were expressed as percentage radical scavenging, and IC_50_ values were calculated from the concentration–response curves. When 50% enzyme inhibition was not reached within the tested concentration range, an IC_50_ value was not calculated.

#### 4.4.2. Glucose Uptake in Cell Culture

A cellular glucose uptake assay was performed to assess whether *P. equisetiforme* extracts or fractions could enhance glucose uptake in insulin-responsive cells. The 3T3-L1 mouse preadipocyte cell line (ATCC^®^ CL-173^TM^; American Type Culture Collection, Manassas, VA, USA) was used as the model. Preadipocytes were serum-starved overnight. The assay was conducted using a commercial 2-deoxyglucose uptake kit (Abcam, Cambridge, UK; catalog no. ab136955) following the manufacturer’s instructions with slight modifications for a 96-well format. Cells were washed with KRPH buffer (Krebs-Ringer phosphate buffer, pH 7.4) containing 2% BSA and incubated in this buffer for 40 min. Treatments were then added to the cells in KRPH buffer. The treatments included: (a) vehicle control, (b) insulin (50 nM; Eli Lilly and Company, Indianapolis, IN, USA) as positive control, (c) plant samples at 50 μg/mL or 100 μg/mL, alone, and (d) plant samples (50 or 100 μg/mL) in the presence of insulin (50 nM). The plant samples tested in this assay were the crude methanol extract (PE-MeOH) and the aqueous extract (PE-H_2_O). After adding treatments, cells were incubated for 2 h at 37 °C. Then 10 mM 2-deoxy-D-glucose (2-DG) was added to each well and incubation continued for 20 min. The uptake of 2-DG into cells was terminated by quickly washing cells with ice-cold PBS three times. Cells were lysed, and intracellular 2-DG-6-phosphate levels were quantified via the kit’s enzymatic colorimetric method at 412 nm. Glucose uptake for each treatment was expressed as pmol per well (control without insulin or sample). Each condition was tested in quadruplicate wells, and the experiment was repeated three independent times. Glucose-uptake data were analyzed separately at each extract concentration using ordinary two-way ANOVA, with insulin exposure (0 or 50 nM) and extract treatment (control, PE-H_2_O, or PE-MeOH) as the two factors. Šídák’s multiple-comparisons test was used to compare each extract treatment with the corresponding control within each insulin condition. Data are presented as mean ± SD from three independent experiments (*n* = 3), and *p* < 0.05 was considered statistically significant [22,23]. Statistical analyses and graphical evaluations were performed using GraphPad Prism version 10.0 (GraphPad Software, Boston, MA, USA).

## 5. Conclusions

This study provides an integrated phytochemical and bioactivity evaluation of *P. equisetiforme* collected from Türkiye, leading to the isolation and identification of seven different secondary metabolites, including epicatechin-3-acetate and tamarixetin, reported here for the first time in the *Polygonum* genus. The coexistence of flavonoid glycosides and catechins is consistent with the plant’s traditional use as a tea and is associated with its antioxidant capacity and inhibition of key metabolic enzymes. Together, these findings support the potential relevance of *P. equisetiforme* for metabolic health, particularly in the context of obesity and diabetes, and highlight its promise as a nutraceutical or functional food component, warranting further in vivo and clinical investigation.

## Figures and Tables

**Table 1:** Total phenolic, flavonoid, and tannin contents of *P. equisetiforme* extracts and fractions. Values are expressed in mg equivalents per g of dry sample (mean of three determinations). GAE: gallic acid equivalents; QE: quercetin equivalents; CE: catechin equivalents.

Sample	Total Phenolics (mg GAE/g)	Total Flavonoids (mg QE/g)	Condensed Tannins (mg CE/g)
PE-MeOH	523 ± 8	103.4 ± 2.5	537.4 ± 10.1
PE-H_2_O	463 ± 5	104.0 ± 1.8	425.4 ± 9.5
Fr. A	127.5 ± 4.2	n.d.	n.d.
Fr. B	549 ± 7	105.6 ± 3.0	821.6 ± 15.3
Fr. C	675 ± 10	103.8 ± 2.1	545.7 ± 11.8
Fr. D	799 ± 9	121.8 ± 4.1	578.4 ± 12.6
Fr. E	835 ± 6	284.6 ± 5.2	862.3 ± 17.9
Fr. F	425.2 ± 6.5	117.2 ± 3.5	900.8 ± 20.5
Fr. G	760.6 ± 8.3	190.0 ± 4.8	955.5 ± 22.0

n.d.: not detected (below limit).

**Table 2:** Enzyme-inhibition activity of *P. equisetiforme* extracts and fractions. IC_50_ values (μg/mL) for inhibition of α-amylase, α-glucosidase, and pancreatic lipase.

Sample	IC_50_ α-Amylase (μg/mL)	IC_50_ α-Glucosidase (μg/mL)	IC_50_ Pancreatic Lipase (μg/mL)
MeOH extract	310.95 ± 9.85	374.04 ± 12.42	519.2 ± 20.51
Water extract	285.0 ± 8.62	362.82 ± 10.91	868.5 ± 30.10
Acarbose	117.73 ± 4.13	118.18 ± 3.98	-
Orlistat	-	-	328.3 ± 11.1

**Table 3:** IC_50_ values of catechin and quercetin in antioxidant and enzyme-inhibitory assays.

Compound	DPPH IC_50_ (µg/mL)	ABTS IC_50_ (µg/mL)	α-Glucosidase IC_50_ (µg/mL)	Pancreatic Lipase IC_50_ (µg/mL)
Catechin	58.73	42.58	<25	56.35 ± 1.26
Quercetin	5.70	3.91	<12.5	18.62 ± 0.20

DPPH and ABTS IC_50_ values were calculated by nonlinear regression of the concentration–response data. Pancreatic lipase results are expressed as mean ± SD values.

**Table 4:** Structures, names and molecular formulas of the compounds obtained from *P. equisetiforme*.

Compound	Abbreviation	Molecular Formula	Molecular Structure
3-*O*-Acetyl-(−)-epicatechin	**PEC-2a**	C_17_H_16_O_7_	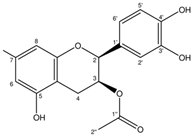
Liquiritin	**PEC-2b**	C_21_H_22_O_9_	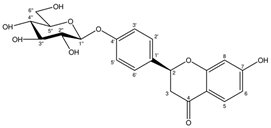
Catechin	**PEE-1**	C_15_H_14_O_6_	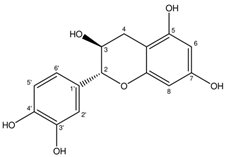
Tamarixetin	**PEE-2**	C_16_H_12_O_7_	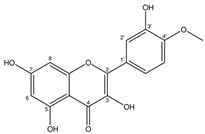
Quercetin	**PEE-3**	C_15_H_10_O_7_	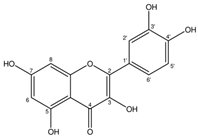
Quercetin-3-O-glucoside (Isoquercitrin)	**PEE-4a**	C_21_H_20_O_12_	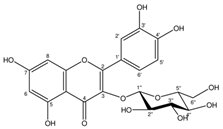
Quercetin-3-O-galactoside (Hyperoside)	**PEE-4b**	C_21_H_20_O_12_	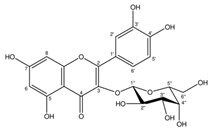

The spectroscopic characterization of the isolated samples is provided below. For samples obtained as mixtures, the signals were assigned to the individual constituents by a combined evaluation of the 1D and 2D NMR spectra.

## Data Availability

The data presented in this study are available within the article and its Supplementary Materials.

## Supplementary Materials

The following supporting information can be downloaded at https://www.mdpi.com/article/10.3390/molecules31183327/s1.

## Abbreviations

The following abbreviations are used in this manuscript:

^13^C NMR	Carbon-13 Nuclear Magnetic Resonance
1D	One-Dimensional
^1^H NMR	Proton Nuclear Magnetic Resonance
2D	Two-Dimensional
2-DG	2-Deoxy-D-glucose
2-DG-6-phosphate	2-Deoxy-D-glucose 6-phosphate
3T3-L1	3T3-L1 mouse preadipocyte cell line (established cell-line name; not a conventional acronym)
A	Absorbance
ABTS	2,2′-Azino-bis(3-ethylbenzothiazoline-6-sulfonic acid)
ABTS•^+^/ABTS^+^·	ABTS radical cation
ANOVA	Analysis of Variance
BSA	Bovine Serum Albumin
CC BY	Creative Commons Attribution
CE	Catechin Equivalents
COSY	Correlation Spectroscopy
CUPRAC	Cupric Ion Reducing Antioxidant Capacity
DI	Deionized
DMSO	Dimethyl Sulfoxide
DNS	3,5-Dinitrosalicylic Acid
DPPH	2,2-Diphenyl-1-picrylhydrazyl
Fr.	Fraction
FRAP	Ferric Reducing Antioxidant Power
GAE	Gallic Acid Equivalents
GLUT4	Glucose Transporter Type 4
HMBC	Heteronuclear Multiple-Bond Correlation
HMQC	Heteronuclear Multiple-Quantum Coherence
HPLC-DAD	High-Performance Liquid Chromatography–Diode Array Detection
HSQC	Heteronuclear Single-Quantum Coherence
HUEF	Hacettepe University Faculty of Pharmacy Herbarium
IC_50_	Half-Maximal Inhibitory Concentration
KRPH	Krebs–Ringer phosphate buffer (as defined in the manuscript)
LC-MS/MS	Liquid Chromatography–Tandem Mass Spectrometry
MeOH	Methanol
MS	Mass Spectrometry
NBT	Nitroblue Tetrazolium
NMR	Nuclear Magnetic Resonance
NO	Nitric Oxide
O_2_•^−^	Superoxide anion radical
PBS	Phosphate-Buffered Saline
PE	*Polygonum equisetiforme*
PEC-2	Mixture isolated from Fraction C
PEC-2a	(-)-Epicatechin-3-acetate
PEC-2b	Liquiritin
PED	P. equisetiforme Fraction D-derived isolate-code prefix
PED-1	(Catechin-containing fraction
PEE	P. equisetiforme Fraction E-derived isolate-code prefix
PEE-1	(+)-Catechin
PEE-2	Tamarixetin
PEE-3	Quercetin
PEE-4	Mixture isolated from Fraction E
PEE-4a	Quercetin-3-O-glucoside (isoquercitrin)
PEE-4b	Quercetin-3-O-galactoside (hyperoside)
PE-H_2_O	Defatted aqueous extract of *Polygonum equisetiforme*
PE-MeOH	*Polygonum equisetiforme* methanolic extract
pNPG	p-Nitrophenyl-α-D-glucopyranoside
QE	Quercetin Equivalents
SD	Standard Deviation
SO	Superoxide anion radical (non-standard abbreviation in the manuscript)
TE	Trolox Equivalents
TEAC	Trolox Equivalent Antioxidant Capacity
TFC	Total Flavonoid Content
TLC	Thin-Layer Chromatography
TPC	Total Phenolic Content
TPTZ	2,4,6-Tris(2-pyridyl)-s-triazine
WHO	World Health Organization
