# Supplementary material for: Isolation of Phenolic Metabolites and Evaluation of the Antioxidant and Metabolic Enzyme-Inhibitory Activities of Polygonum equisetiforme Sm
Source: Molecules. 2026 Sep 19;31(18):3327. doi: 10.3390/molecules31183327 (PMC13610121; doi:10.3390/molecules31183327)
Supplement: Supplementary file 1 [file molecules-31-03327-s001.zip › molecules-4510605-supplementary.pdf]

Supplementary Spectroscopic Data for Isolated Compounds

PEC-2: an inseparable mixture of 3-O-acetyl-(−)-epicatechin (PEC-2a) and liquiritin (PEC-2b)

Sample-code note. The raw spectra were acquired under the internal laboratory code PEC-7. The same sample is designated PEC-2 in the manuscript and in the figure captions below; the original raw-data label remains visible to preserve traceability.

Figure S1. ^{1}H NMR spectrum of PEC-2 (internal raw-data code PEC-7) in DMSO-d_6.

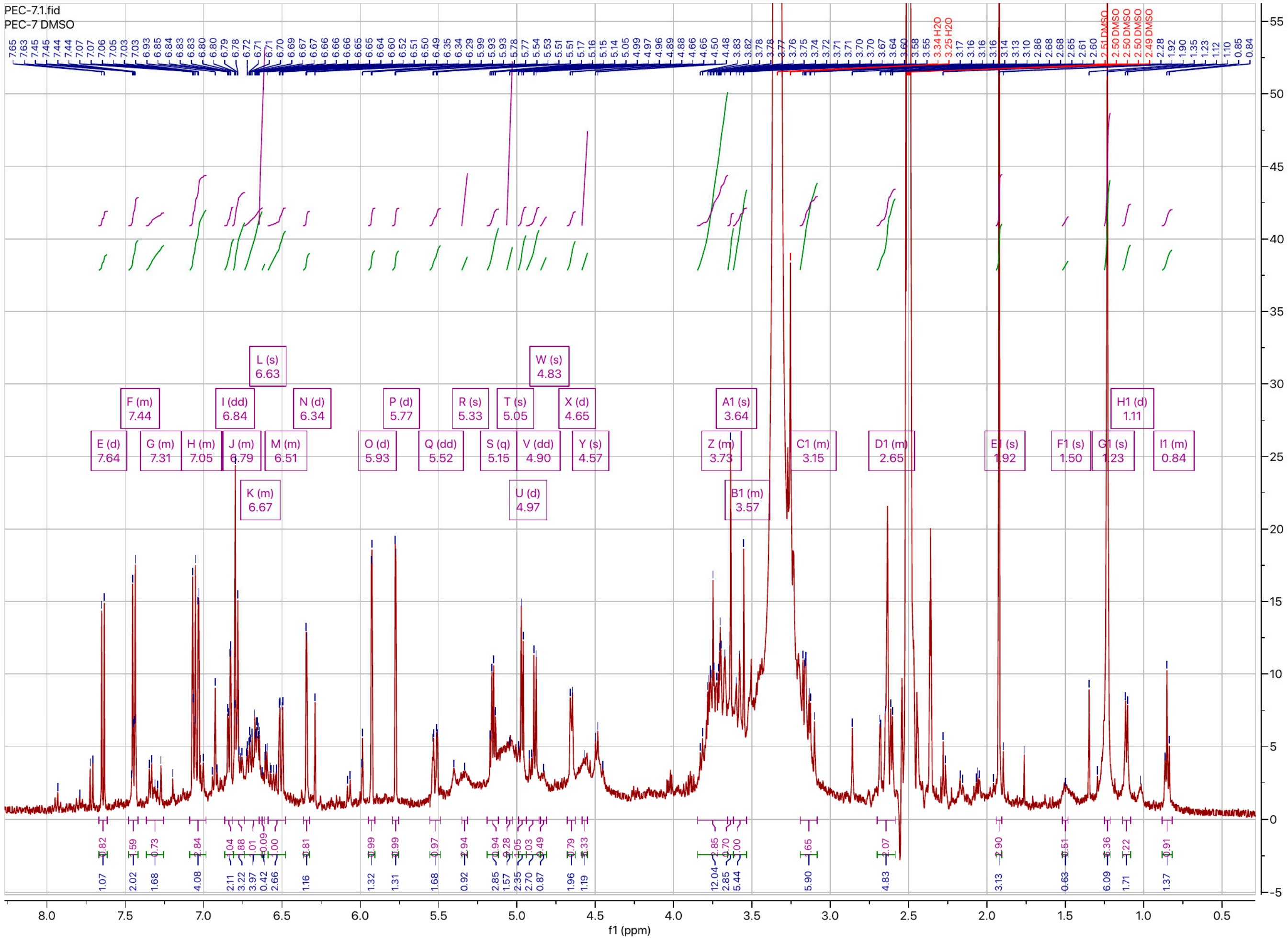

Diagnostic assignments: PEC-2a: δH 5.15 (H-3) and 1.92 (s, 3H, $OCOCH_3$); PEC-2b: δH 5.52 (H-2), 4.97 (H-1″), 3.15 (H-3a), and 2.65 (H-3b).

Figure S2. ^{13}C NMR/APT spectrum of PEC-2 (internal raw-data code PEC-7) in DMSO-d_6.

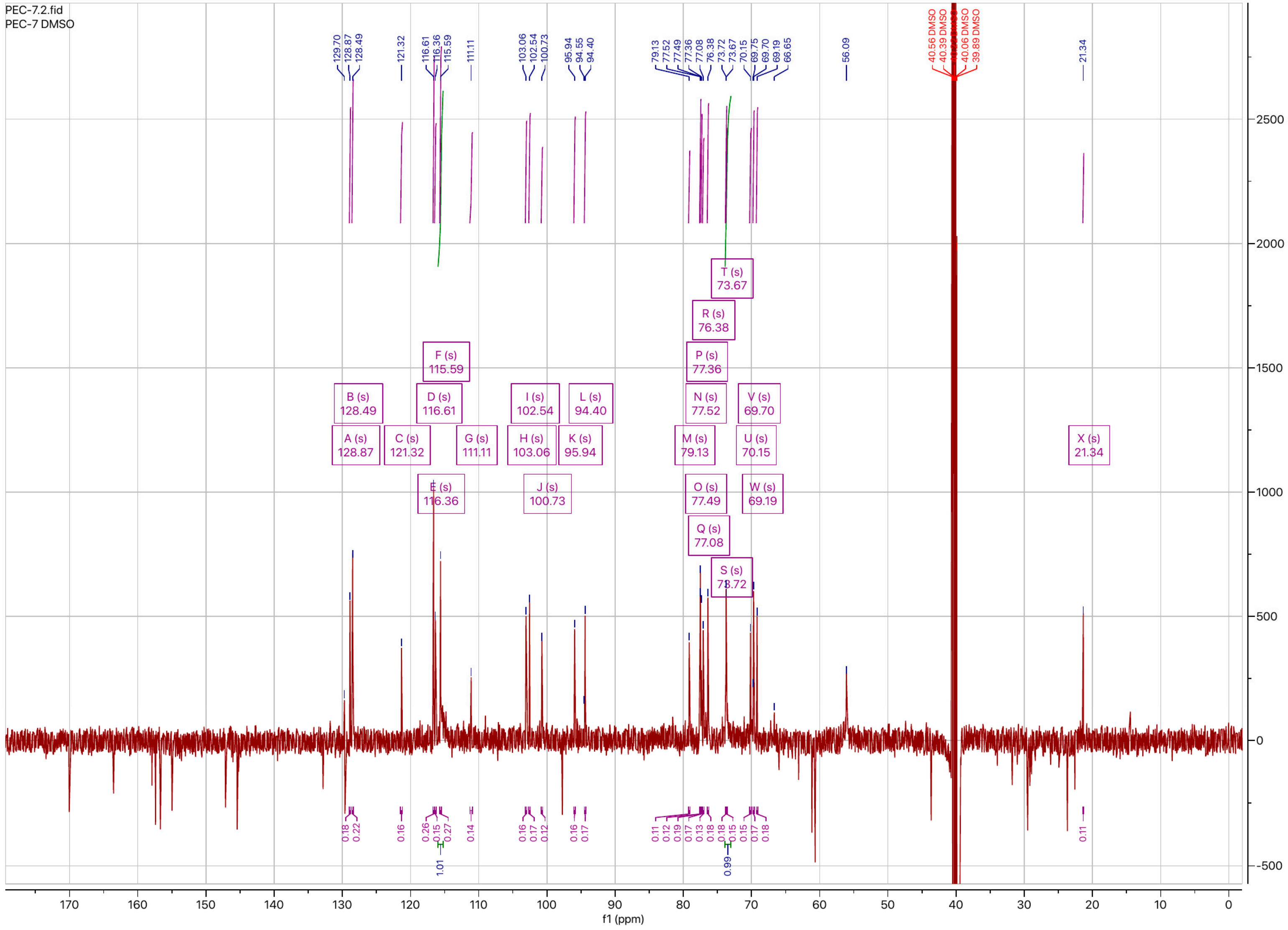

Diagnostic assignments: PEC-2a: δC 170.13 (acetate carbonyl), 69.20 (C-3), and 21.34 ($OCOCH_3$); PEC-2b: δC 190.4 (C-4), 100.3 (C-1″), and 60.7–79.1 (glucosyl carbons). Low-intensity quaternary/carbonyl signals are more clearly supported in the HMBC spectrum.

Figure S3. HSQC spectrum of PEC-2 (internal raw-data code PEC-7) in DMSO-d_6.

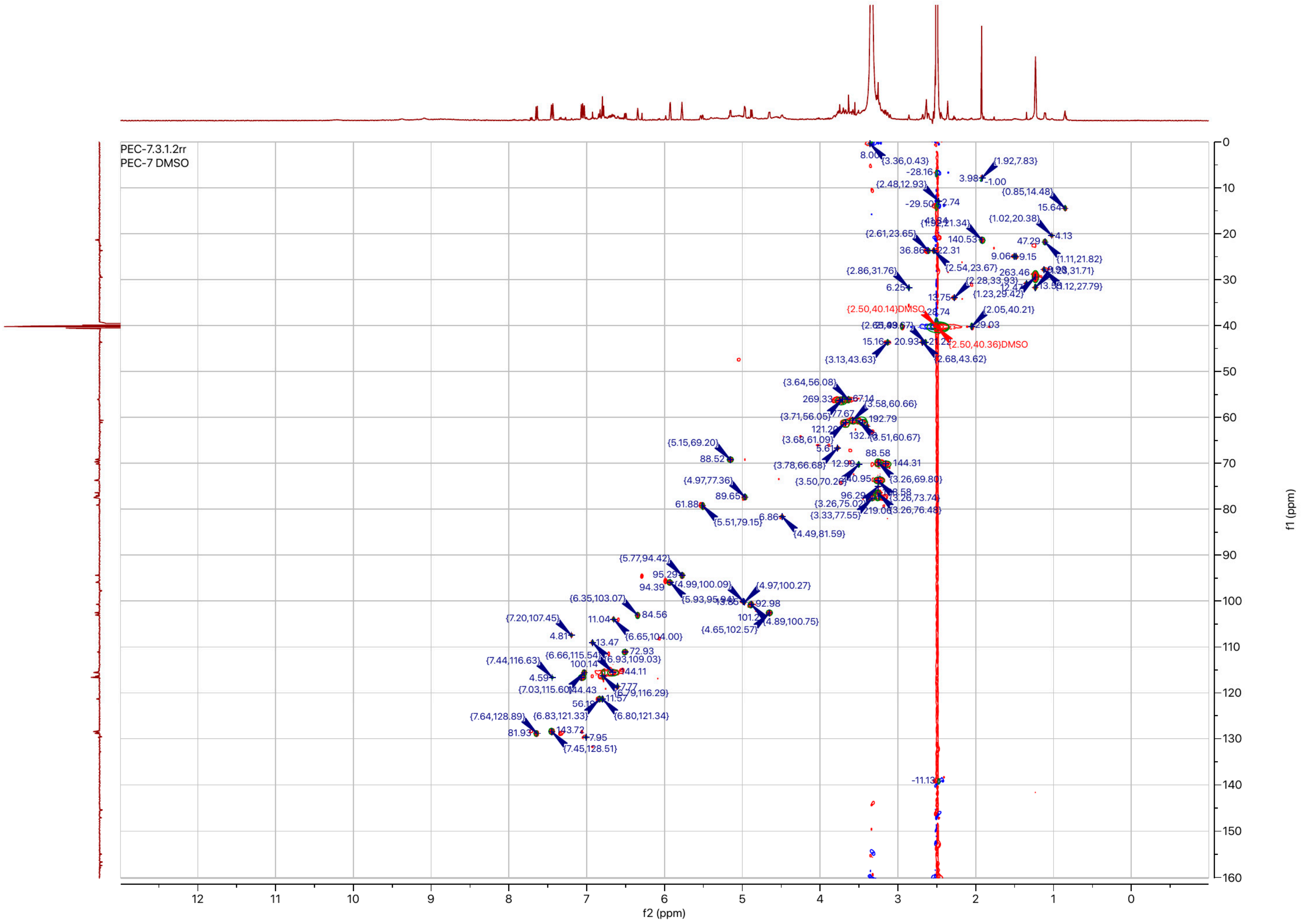

Key correlations: δH/δC 5.15/69.20 (H-3/C-3 of PEC-2a) and approximately 4.97/100.3 (H-1″/C-1″ of PEC-2b), together with correlations for the oxygenated sugar methines and the flavonoid aromatic CH signals.

Figure S4. HMBC spectrum of PEC-2 (internal raw-data code PEC-7) in DMSO-d_6.

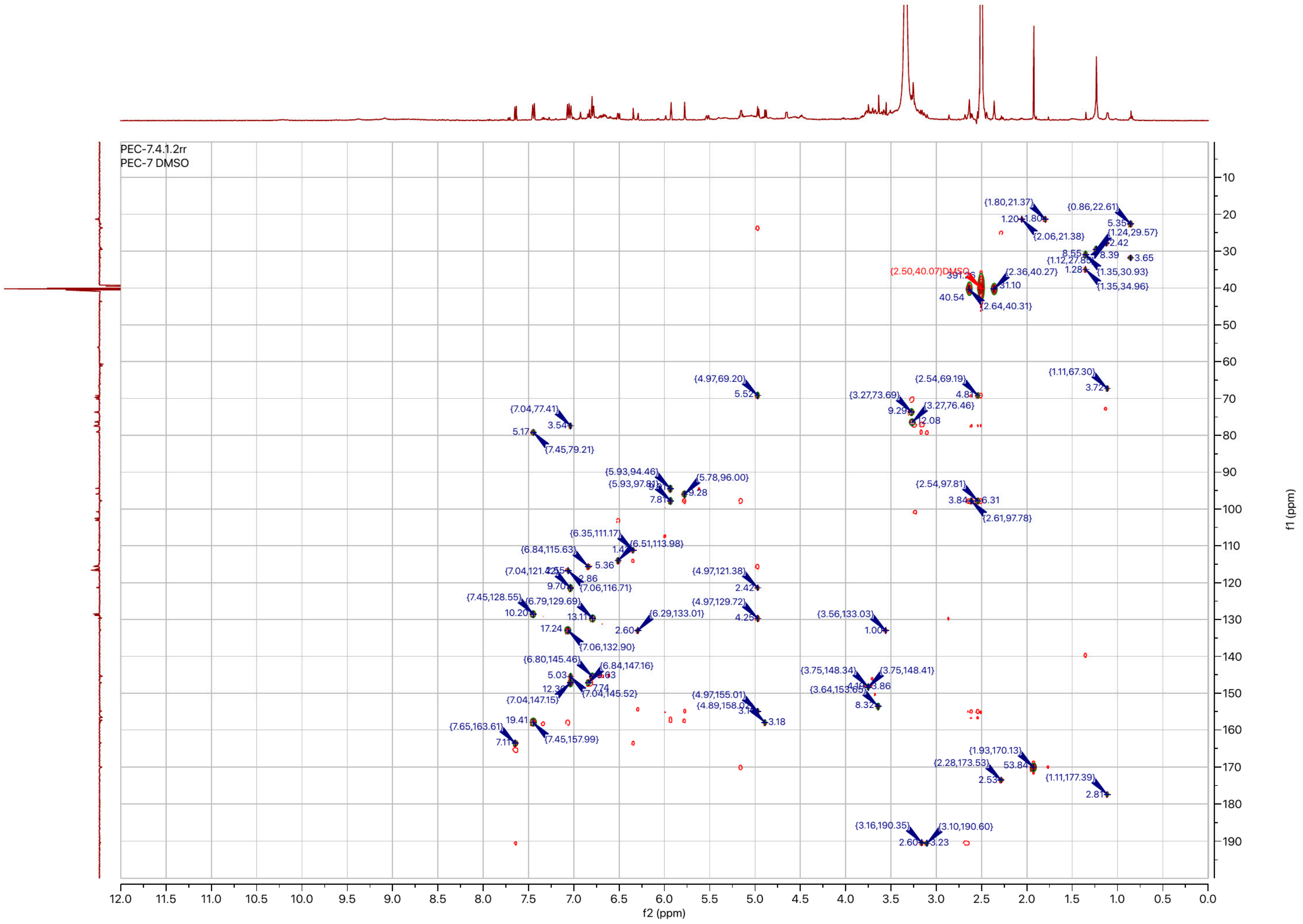

Key long-range correlations: δH 1.93 ($OCOCH_3$) → δC 170.13 (acetate carbonyl), supporting the O-acetyl group of PEC-2a; H-3a/H-3b → C-4 (approximately δC 190.4), supporting the flavanone skeleton of PEC-2b; and H-1″ (δH 4.97) → C-4′ (approximately δC 158.0), supporting 4′-O-glucosylation in liquiritin.

Figure S5. Positive-ion full-scan ESI mass spectrum of PEC-2 (internal raw-data code PEC-7).

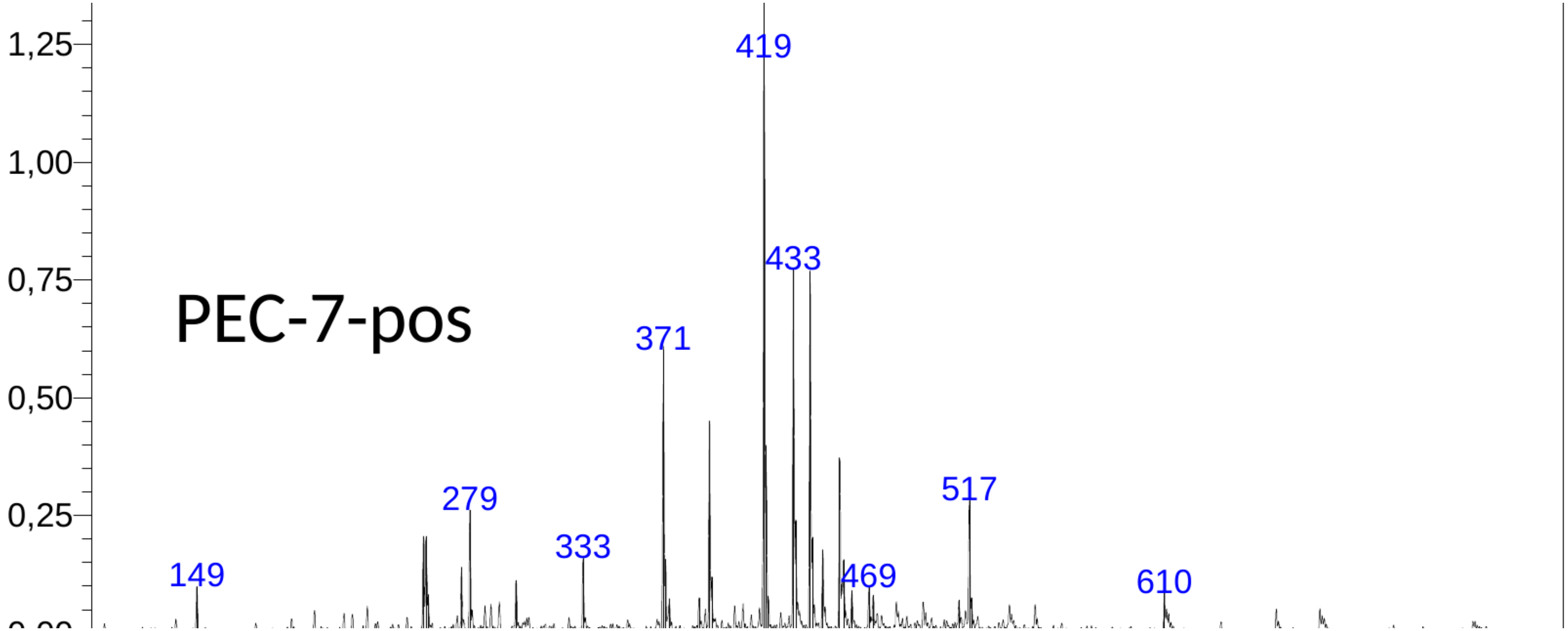

Relevant ions: m/z 333, consistent with $[M + H]^+$ of 3-O-acetyl-(−)-epicatechin ($C_{17}H_{16}O_7$; nominal molecular mass 332), and m/z 419, consistent with $[M + H]^+$ of liquiritin ($C_{21}H_{22}O_9$; nominal molecular mass 418).

Supplementary Spectroscopic Data for Catechin (PEE-1)

Sample-code note. The raw spectra were acquired under the internal laboratory code PEE-2. The same sample is designated PEE-1 in the manuscript and in the figure captions below; the original raw-data label remains visible to preserve traceability.

Figure S6. ^{1}H NMR spectrum of catechin (PEE-1; internal raw-data code PEE-2) in CD_3OD.

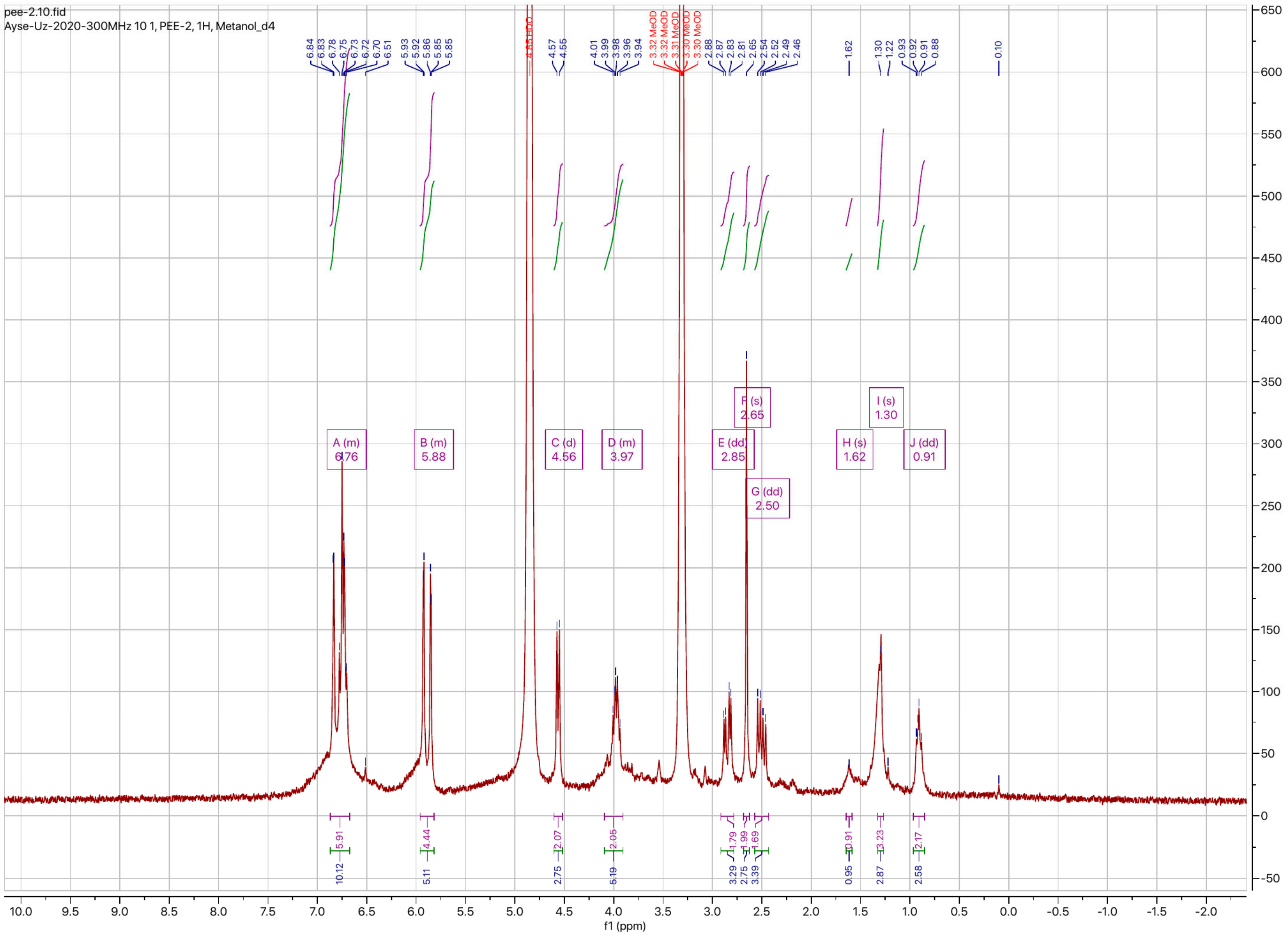

Diagnostic assignments: δH 6.84 (H-2′), 6.76 (H-5′), 6.72 (H-6′), 5.93 (H-8), 5.86 (H-6), 4.56 (H-2), 3.97 (H-3), 2.85 (H-4a), and 2.50 (H-4b). The H-2 doublet (J approximately 7.3 Hz) is consistent with the trans H-2/H-3 relationship of catechin.

Figure S7. Selected COSY region of catechin (PEE-1; internal raw-data code PEE-2) in CD_3OD.

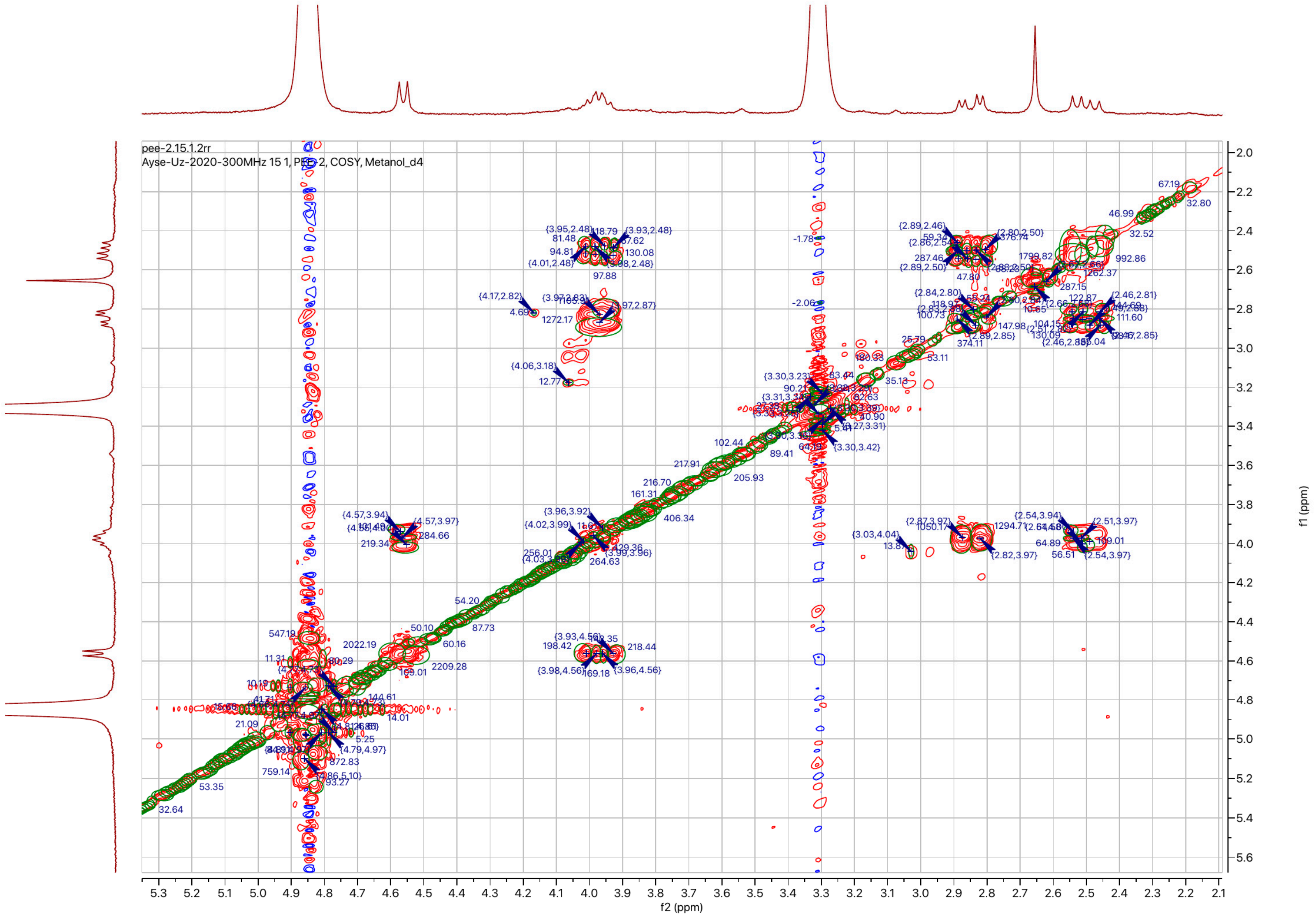

Key correlations: H-2/H-3 and H-3/H-4a,H-4b define the C-ring proton spin system. The observed coupling pattern and the relatively large H-2/H-3 coupling support the catechin rather than epicatechin relative configuration.

Figure S8. HSQC spectrum of catechin (PEE-1; internal raw-data code PEE-2) in CD_3OD.

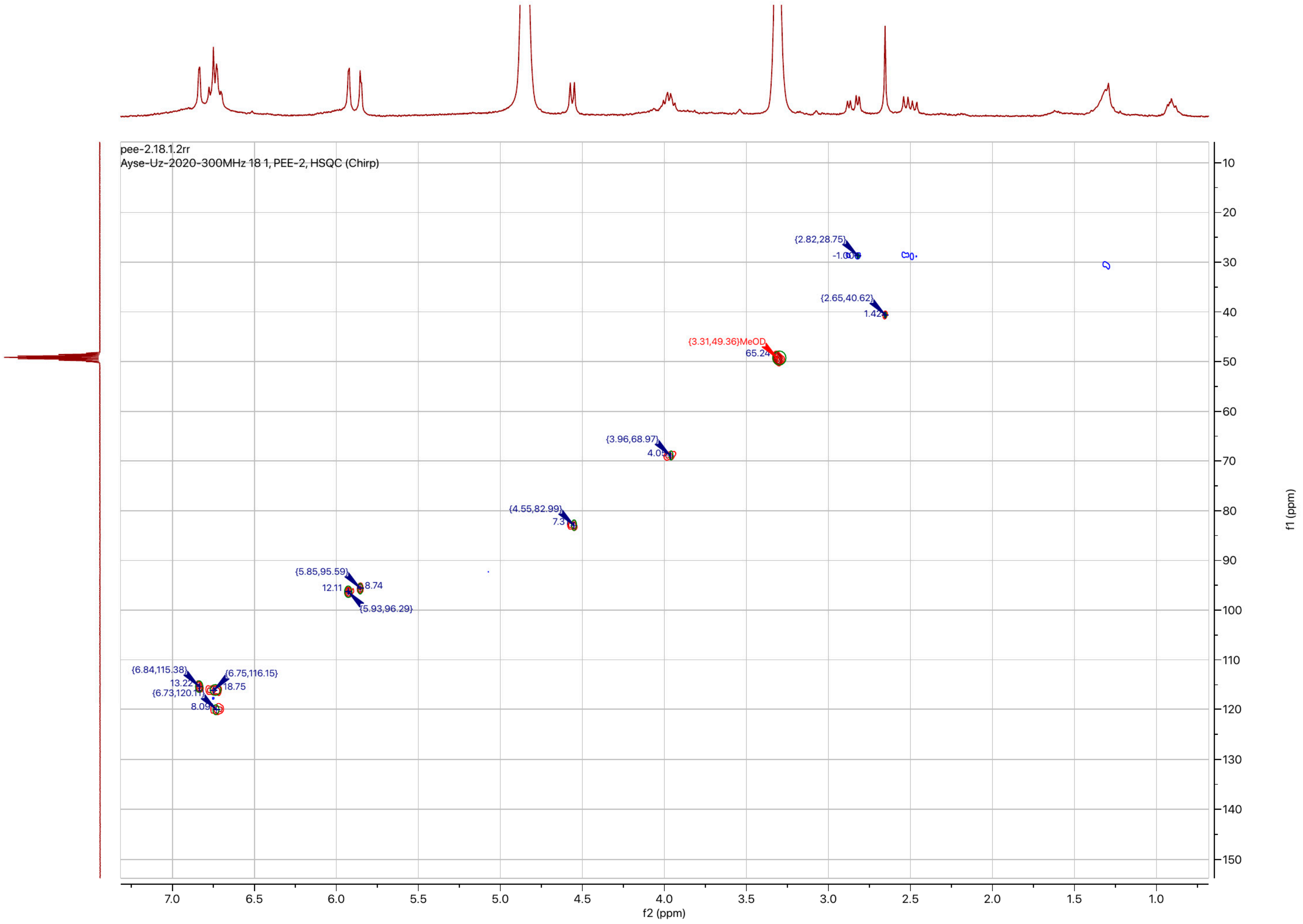

Key correlations: δH/δC 4.55/82.99 (H-2/C-2), 3.96/68.97 (H-3/C-3), 5.93/96.29 (H-8/C-8), 5.85/95.59 (H-6/C-6), 6.84/115.38 (H-2′/C-2′), and 6.75/116.15 (H-5′/C-5′).

Figure S9. Positive-ion full-scan ESI mass spectrum of catechin (PEE-1).

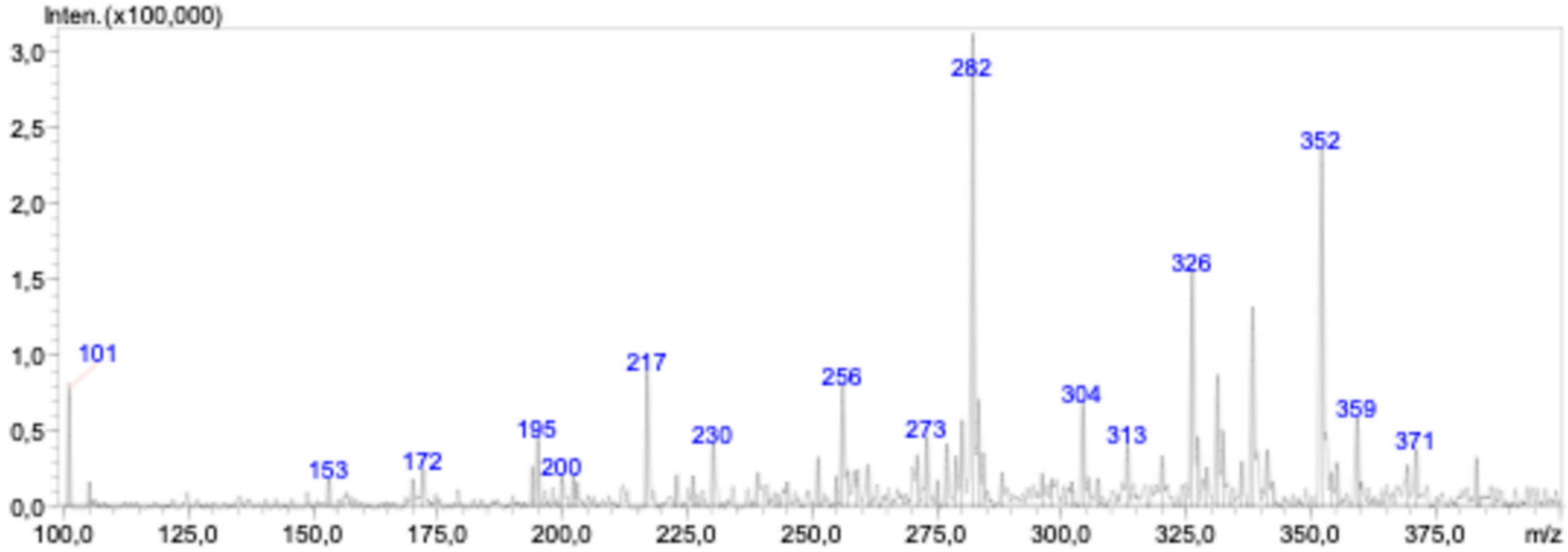

Relevant ion: m/z 313, consistent with the sodium adduct $[M + Na]^+$ of catechin ($C_{15}H_{14}O_6$; nominal molecular mass 290). The full-scan mass spectrum supports the molecular mass.

Supplementary Spectroscopic Data for Tamarixetin (PEE-2)

Sample-code note. The raw spectra were acquired under the internal laboratory code PEE-9. The same sample is designated PEE-2 in the manuscript and in the figure captions below; the original raw-data label remains visible to preserve traceability.

Figure S10. ^{1}H NMR spectrum of tamarixetin (PEE-2; internal raw-data code PEE-9) in CD_3OD.

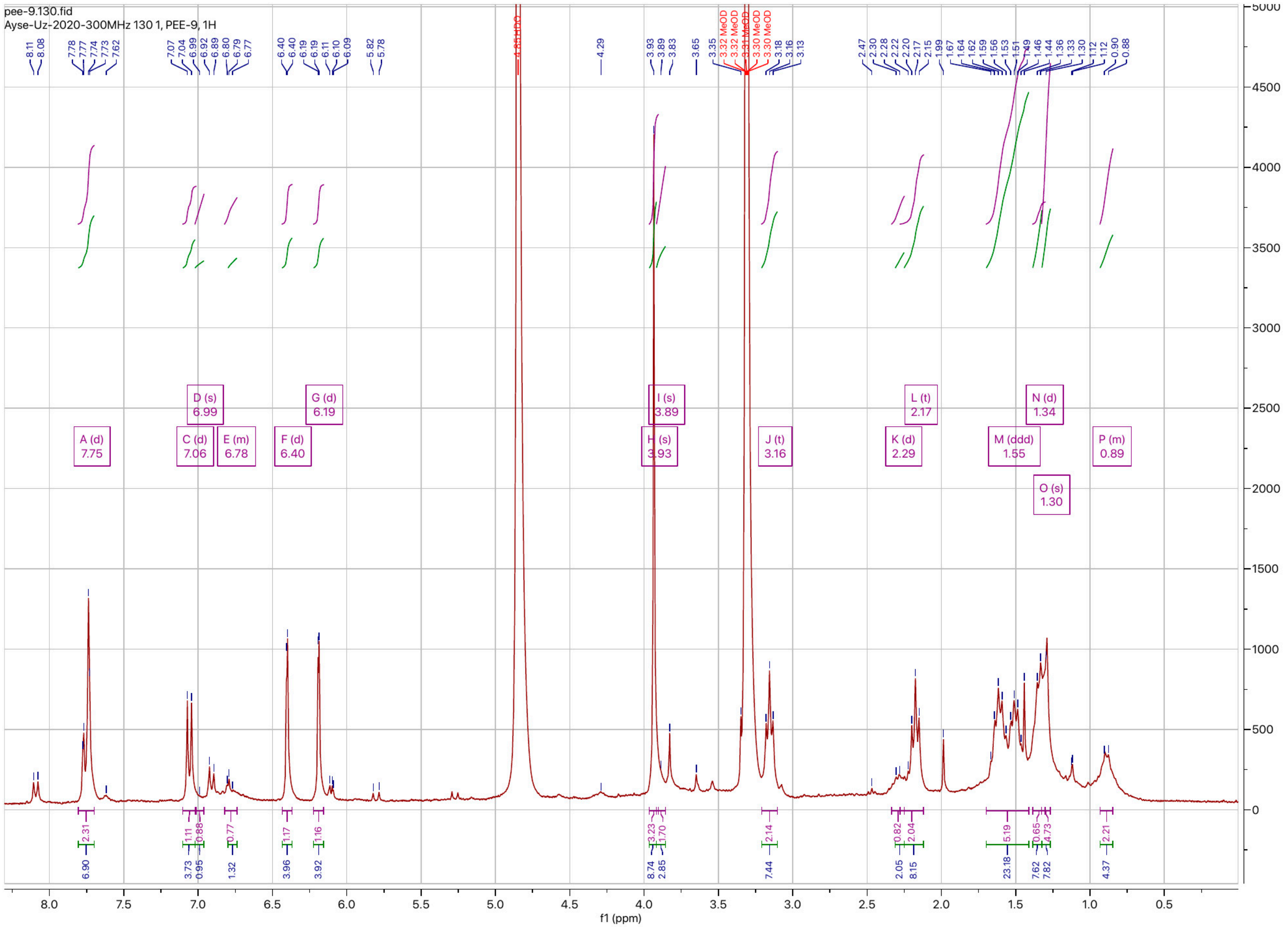

Diagnostic assignments: δH 7.75 (H-6′), 7.74 (H-2′), 7.07 (H-5′), 6.40 (H-8), 6.19 (H-6), and 3.93–3.94 (s, 3H, 4′-OCH_3). The 1,2,4-trisubstituted B-ring pattern, the pair of meta-coupled A-ring protons, and the methoxy singlet support a monomethylated quercetin derivative.

Figure S11. Selected aromatic COSY region of tamarixetin (PEE-2; internal raw-data code PEE-9) in CD_3OD.

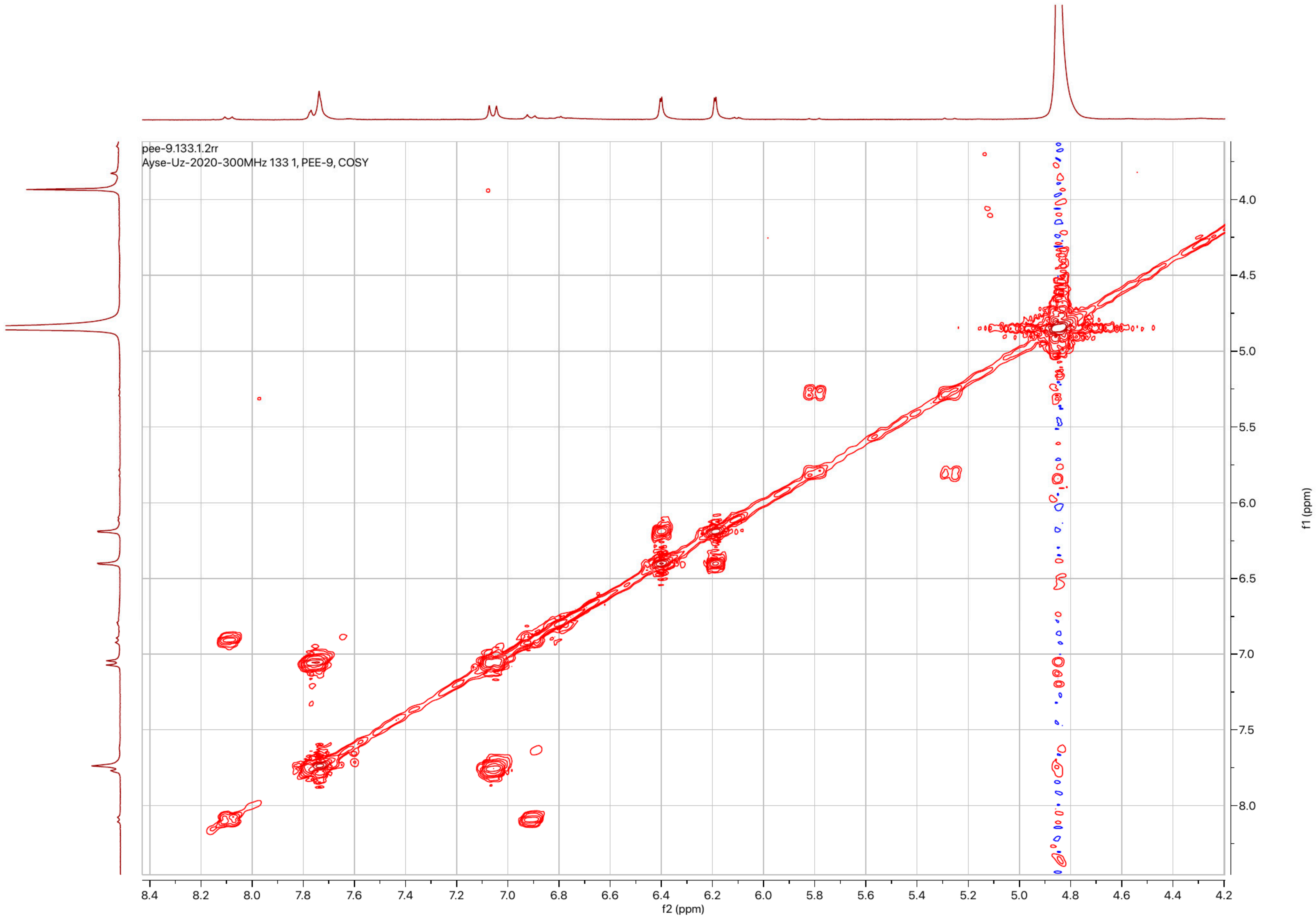

Key correlation: The H-5′/H-6′ cross-peak supports the contiguous ortho-coupled proton pair of the 1,2,4-trisubstituted B ring. Only diagnostically relevant cross-peaks are used for the assignment.

Figure S12. HSQC spectrum of tamarixetin (PEE-2; internal raw-data code PEE-9) in CD_3OD.

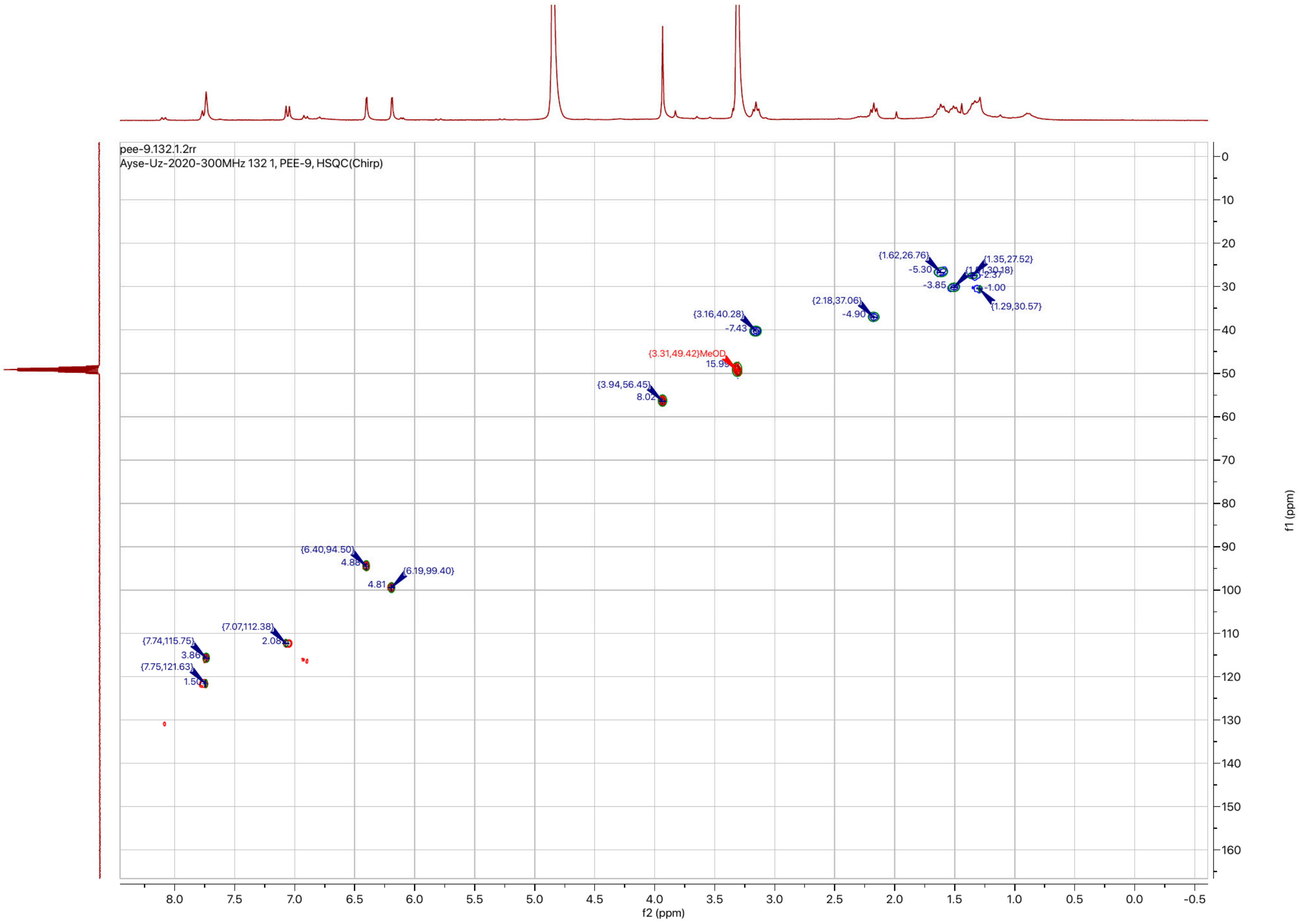

Key correlations: δH/δC 7.75/121.63 (H-6′/C-6′), 7.74/115.75 (H-2′/C-2′), 7.07/112.38 (H-5′/C-5′), 6.40/94.50 (H-8/C-8), 6.19/99.40 (H-6/C-6), and 3.94/56.45 (4′-OCH_3).

Figure S13. HMBC spectrum of tamarixetin (PEE-2; internal raw-data code PEE-9) in CD_3OD.

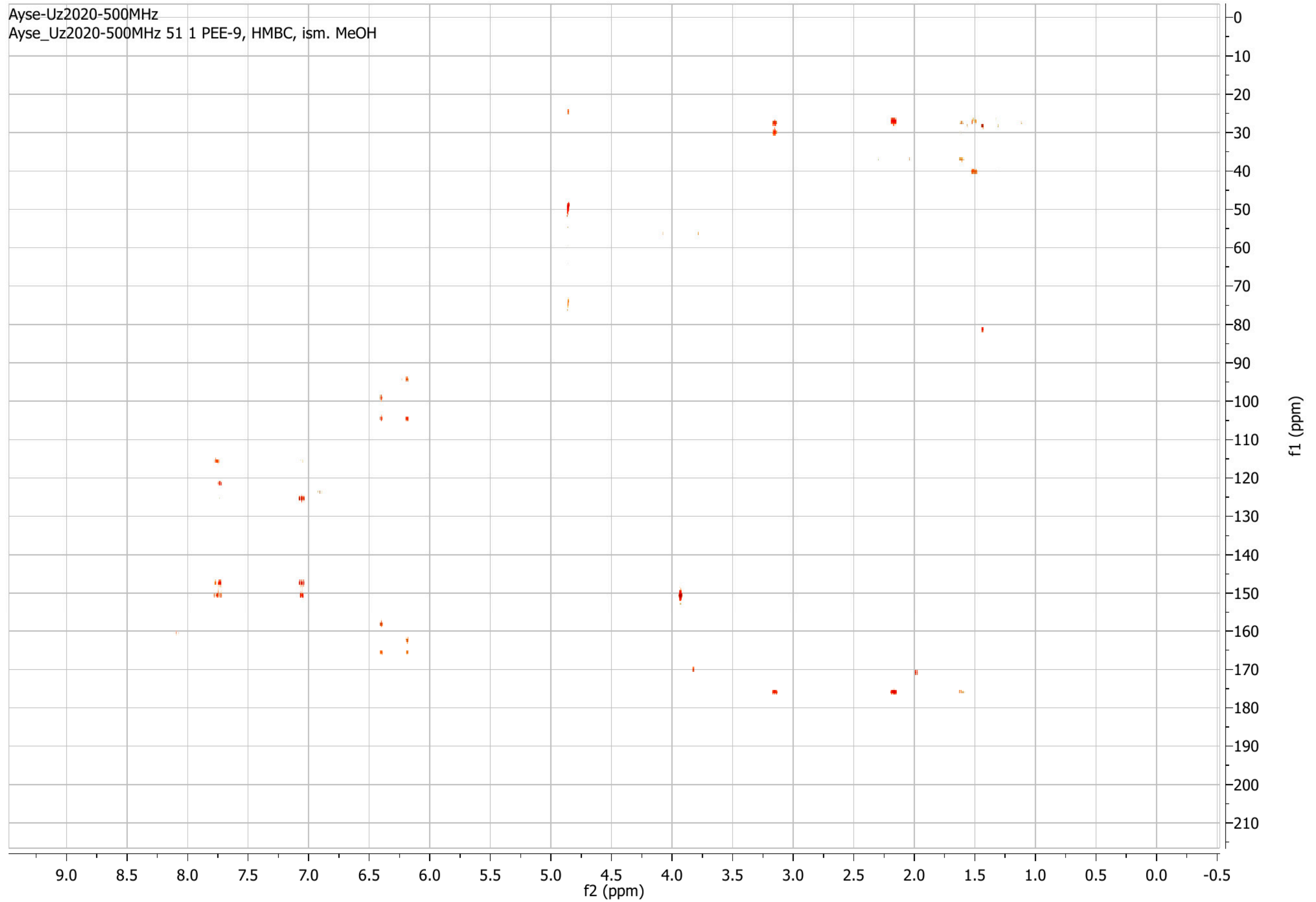

Key long-range correlation: The methoxy singlet at δH approximately 3.94 shows a long-range correlation with the oxygenated aromatic carbon C-4′ (δC approximately 150.8), supporting 4′-O-methyl substitution.

Figure S14. Positive-ion full-scan ESI mass spectrum of tamarixetin (PEE-2; internal raw-data code PEE-9).

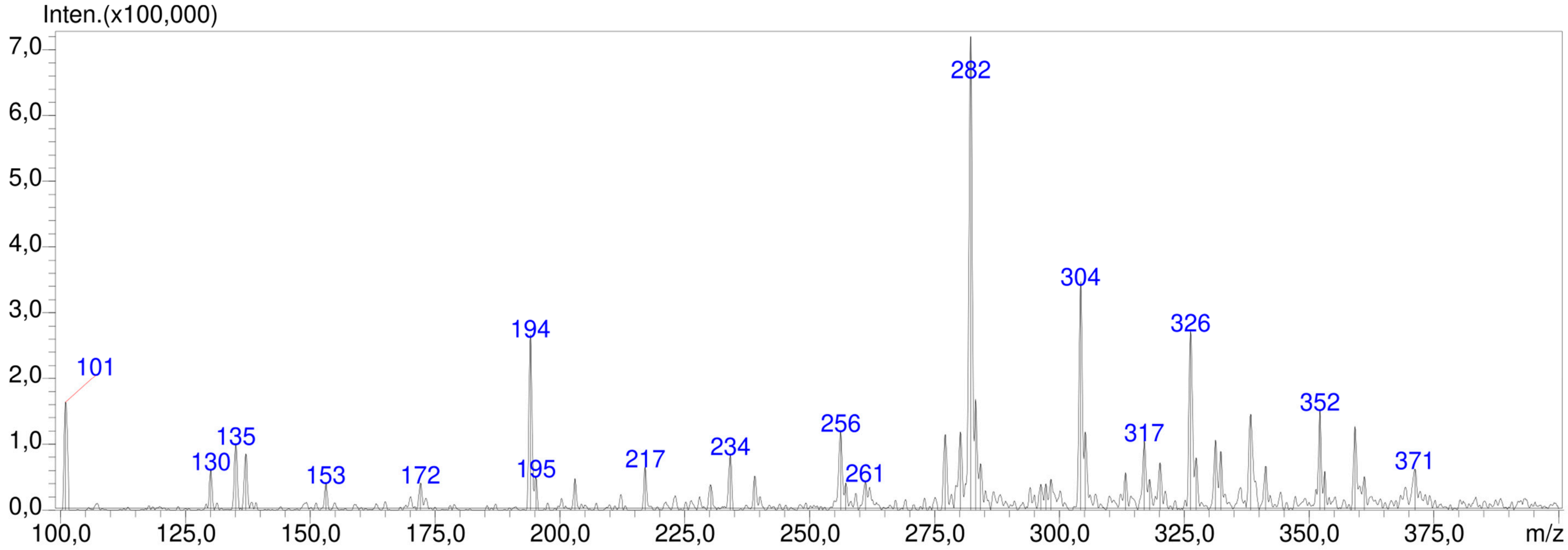

Relevant ion: m/z 317, consistent with $[M + H]^+$ of tamarixetin ($C_{16}H_{12}O_7$; nominal molecular mass 316).

Supplementary Spectroscopic Data for Quercetin (PEE-3)

Sample-code note. The raw spectra were acquired under the internal laboratory code PEE-8a. The same sample is designated PEE-3 in the manuscript and in the figure captions below; the original raw-data label remains visible to preserve traceability.

Figure S15. ^1H NMR spectrum of quercetin (PEE-3; internal raw-data code PEE-8a) in DMSO-d_6.

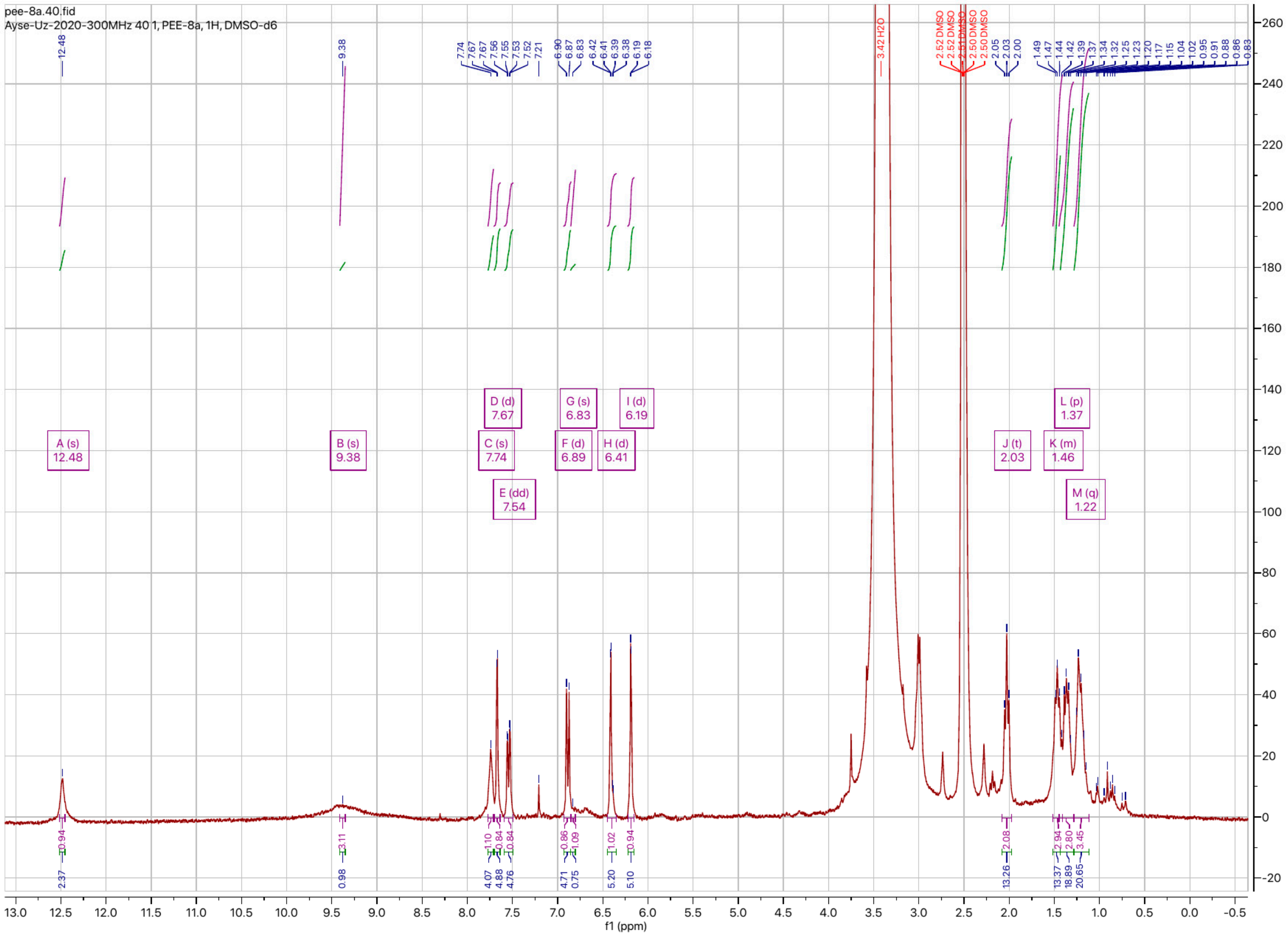

Diagnostic assignments: δH 12.48 (s, 5-OH), 7.67 (d, J approximately 2.1 Hz, H-2′), 7.54 (dd, J approximately 8.5, 2.1 Hz, H-6′), 6.89 (d, J approximately 8.5 Hz, H-5′), 6.41 (d, J approximately 2.0 Hz, H-8), and 6.19 (d, J approximately 2.0 Hz, H-6). The ABX-type B-ring pattern and the two meta-coupled A-ring protons are characteristic of quercetin.

Figure S16. Selected aromatic COSY region of quercetin (PEE-3; internal raw-data code PEE-8a) in DMSO-d_6.

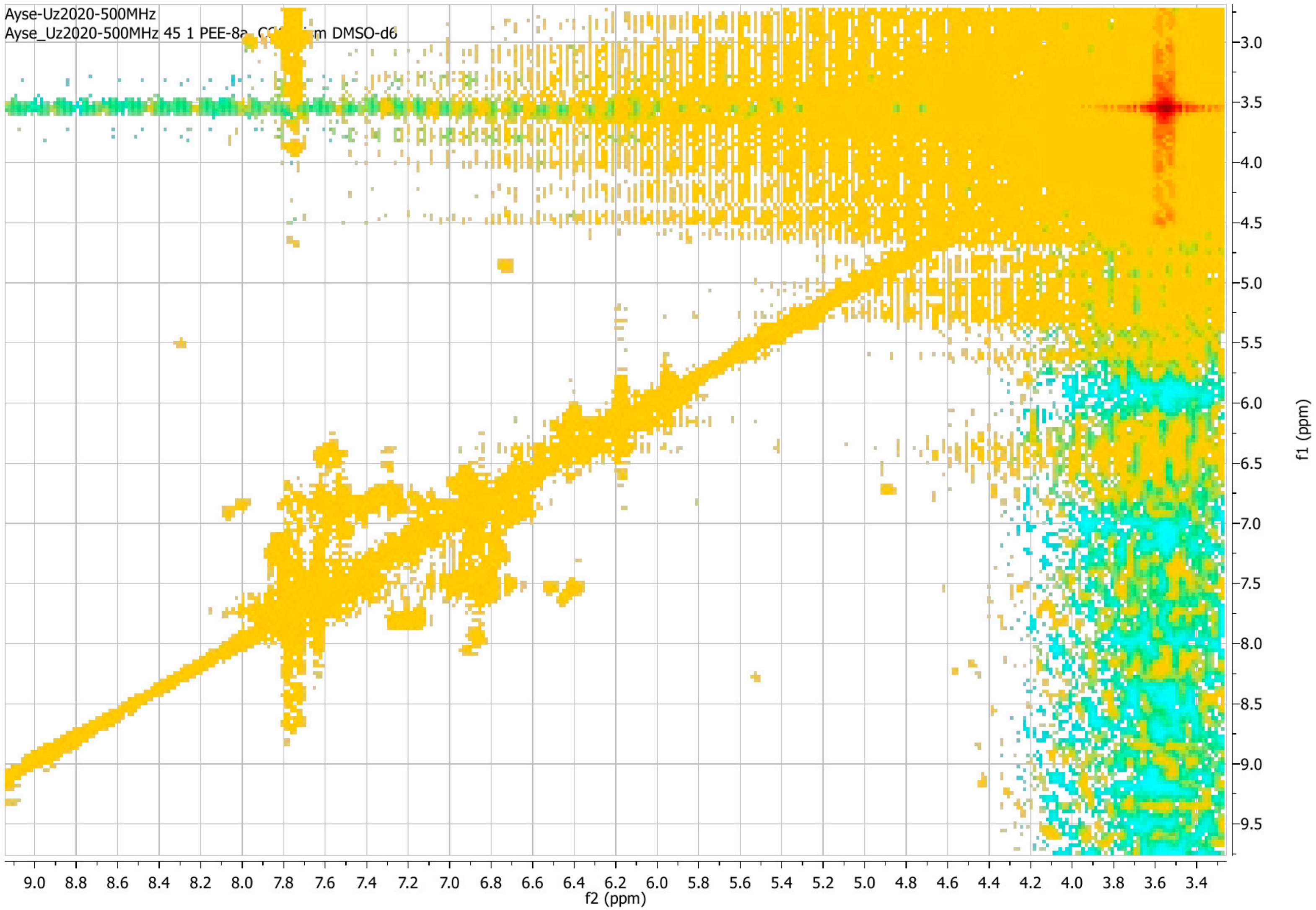

Key correlation: The H-5′/H-6′ cross-peak supports the ortho-coupled proton pair of the 1,2,4-trisubstituted B ring. The isolated meta-coupled A-ring signals at H-6 and H-8 do not require a strong COSY cross-peak for assignment.

Figure S17. Negative-ion full-scan ESI mass spectrum of quercetin (PEE-3; internal raw-data code PEE-8a).

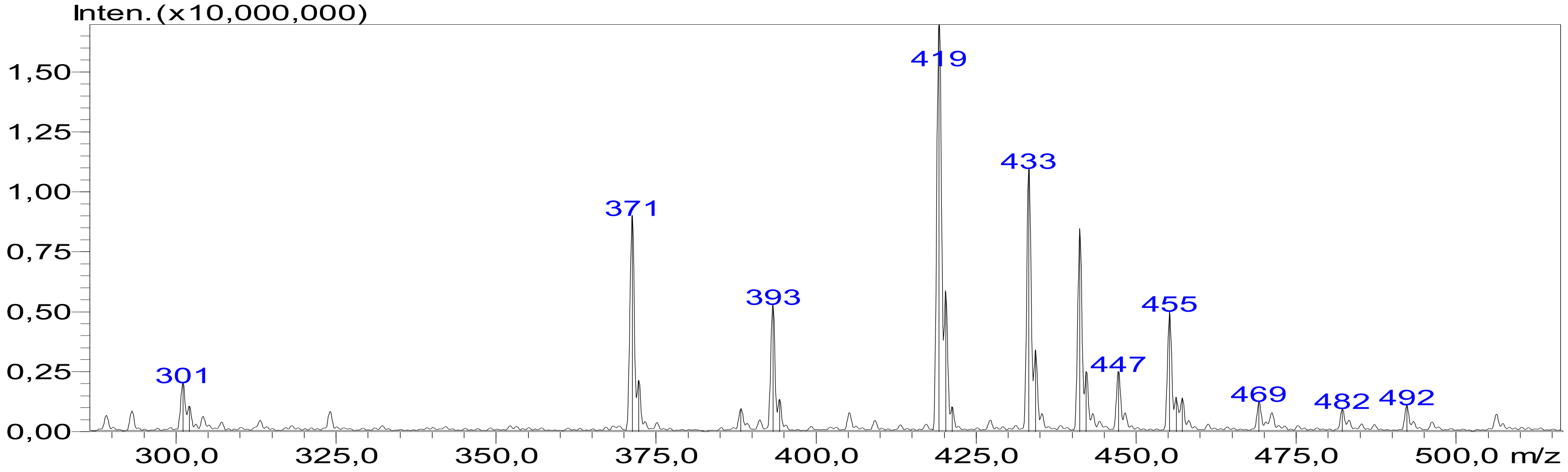

Relevant ion: *m/z* 301, consistent with the deprotonated molecular ion $[M - H]^-$ of quercetin ($C_{15}H_{10}O_7$; calcd for $C_{15}H_9O_7^-$, 301.0354).

Supplementary Spectroscopic Data for PEE-4a/PEE-4b

Sample-code note. The inseparable mixture was acquired under the internal laboratory code PEE-11. Its constituents are designated PEE-4a, quercetin-3-O-glucoside (isoquercitrin), and PEE-4b, quercetin-3-O-galactoside (hyperoside), in the manuscript and in the figure captions below; the original raw-data label remains visible to preserve traceability.

Figure S18. ^{1}H NMR spectrum of the PEE-4a/PEE-4b mixture (internal raw-data code PEE-11) in DMSO-d_6.

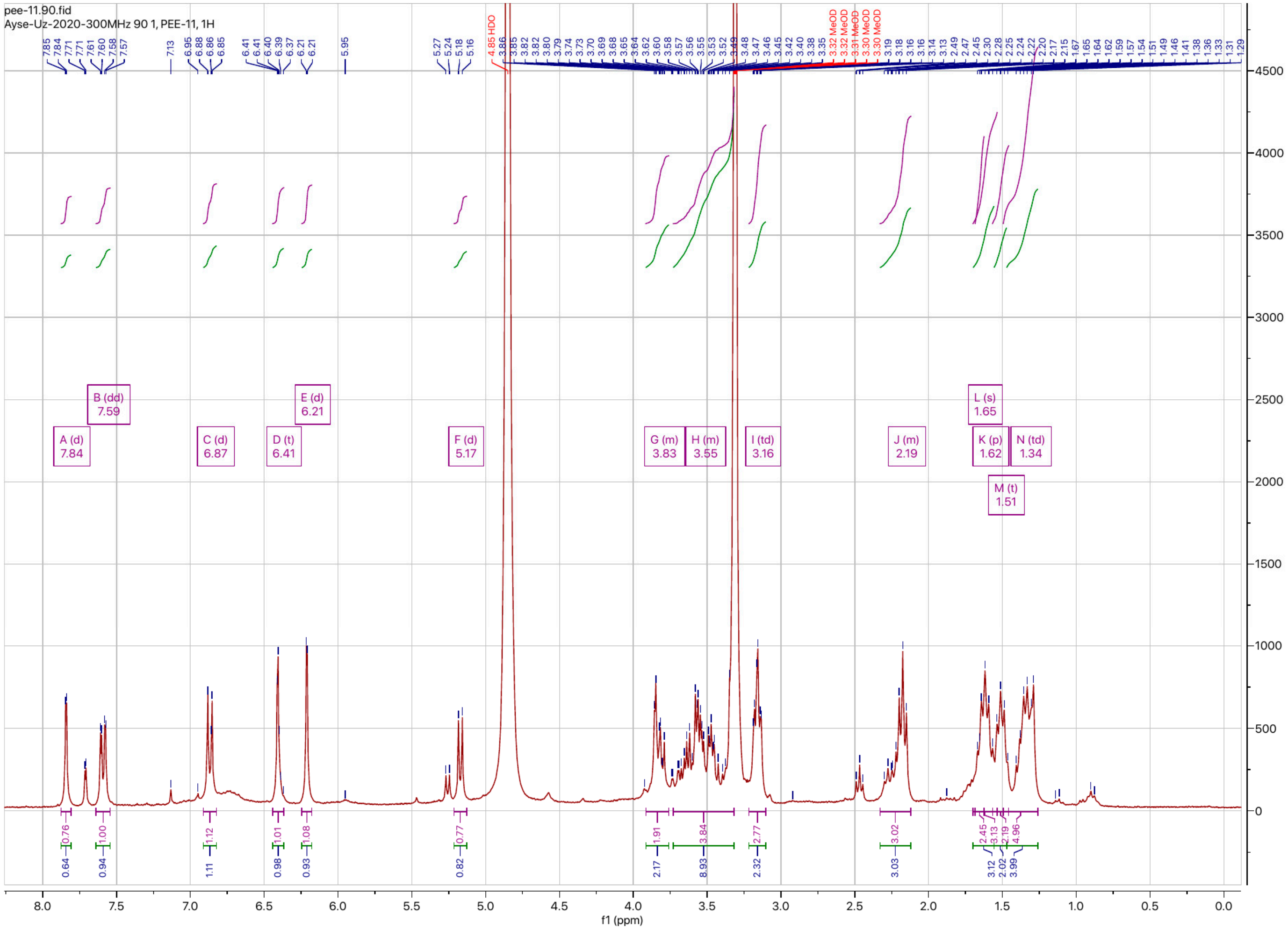

Diagnostic assignments: The common quercetin nucleus showed δH 7.84/7.71 (H-2′), 7.59 (H-6′), 6.87 (H-5′), 6.41 (H-8), and 6.21 (H-6). Two closely spaced anomeric proton signals at approximately δH 5.26 (d, J approximately 7.6 Hz; glucosyl H-1″ of PEE-4a) and 5.17 (d, J approximately 7.7 Hz; galactosyl H-1″ of PEE-4b) support the presence of two β-configured quercetin 3-O-hexosides. The relatively uniform large sugar-proton couplings support the glucopyranosyl residue of PEE-4a, whereas the small H-3″/H-4″ coupling (approximately 3.0 Hz) supports the galactopyranosyl residue of PEE-4b. Sugar resonances overlap extensively between δH approximately 3.2 and 3.9.

Figure S19. COSY spectrum of the PEE-4a/PEE-4b mixture (internal raw-data code PEE-11) in DMSO-d_6.

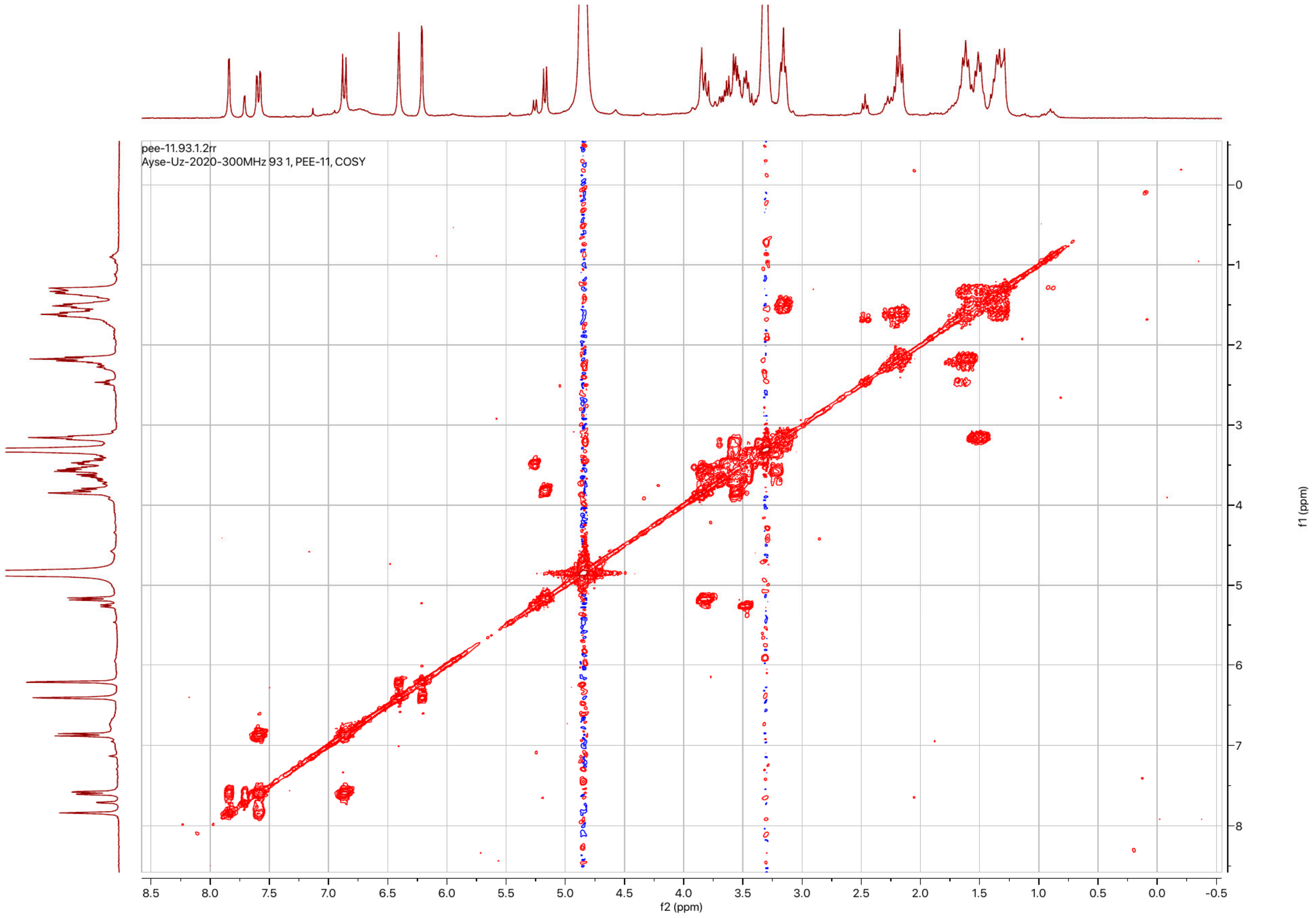

Key correlations: The H-5′/H-6′ correlation supports the 1,2,4-trisubstituted B ring of the quercetin nucleus.

Figure S20. HSQC spectrum of the PEE-4a/PEE-4b mixture (internal raw-data code PEE-11) in DMSO-d_6.

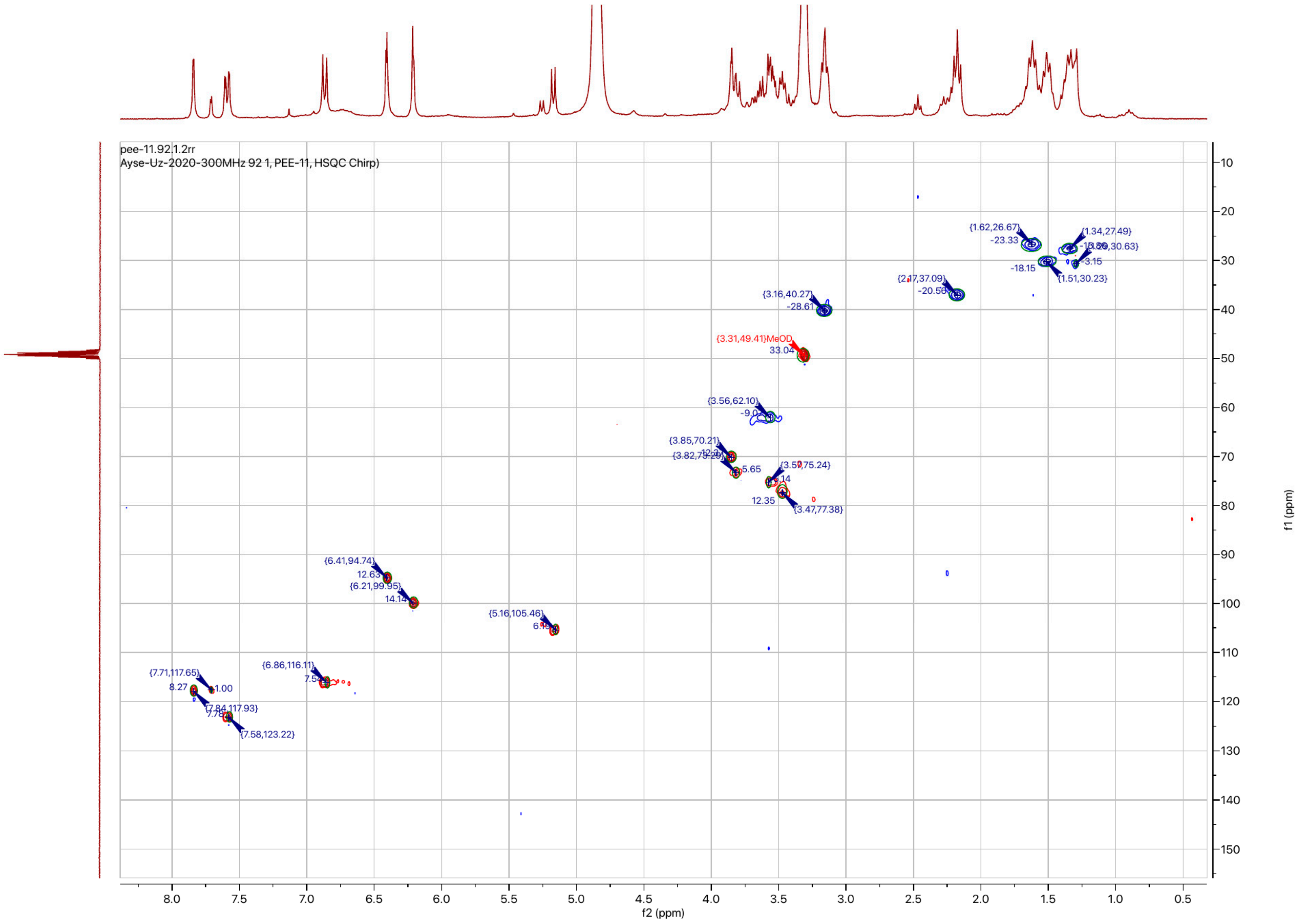

Key correlations: The correlation at approximately δH/δC 5.16/105.46 is assigned to galactosyl H-1″/C-1″ of PEE-4b; the neighboring anomeric correlation near δH 5.26 is attributed to glucosyl H-1″/C-1″ of PEE-4a. Diagnostic quercetin correlations include 7.84/117.93 and 7.58/123.22 for the B ring, 6.41/94.74 (H-8/C-8), and 6.21/99.95 (H-6/C-6). The sugar-region correlations between δH approximately 3.4–3.9 and δC approximately 62–78 support O-linked hexose residues.

Figure S21. HMBC spectrum of the PEE-4a/PEE-4b mixture (internal raw-data code PEE-11) in DMSO-d_6.

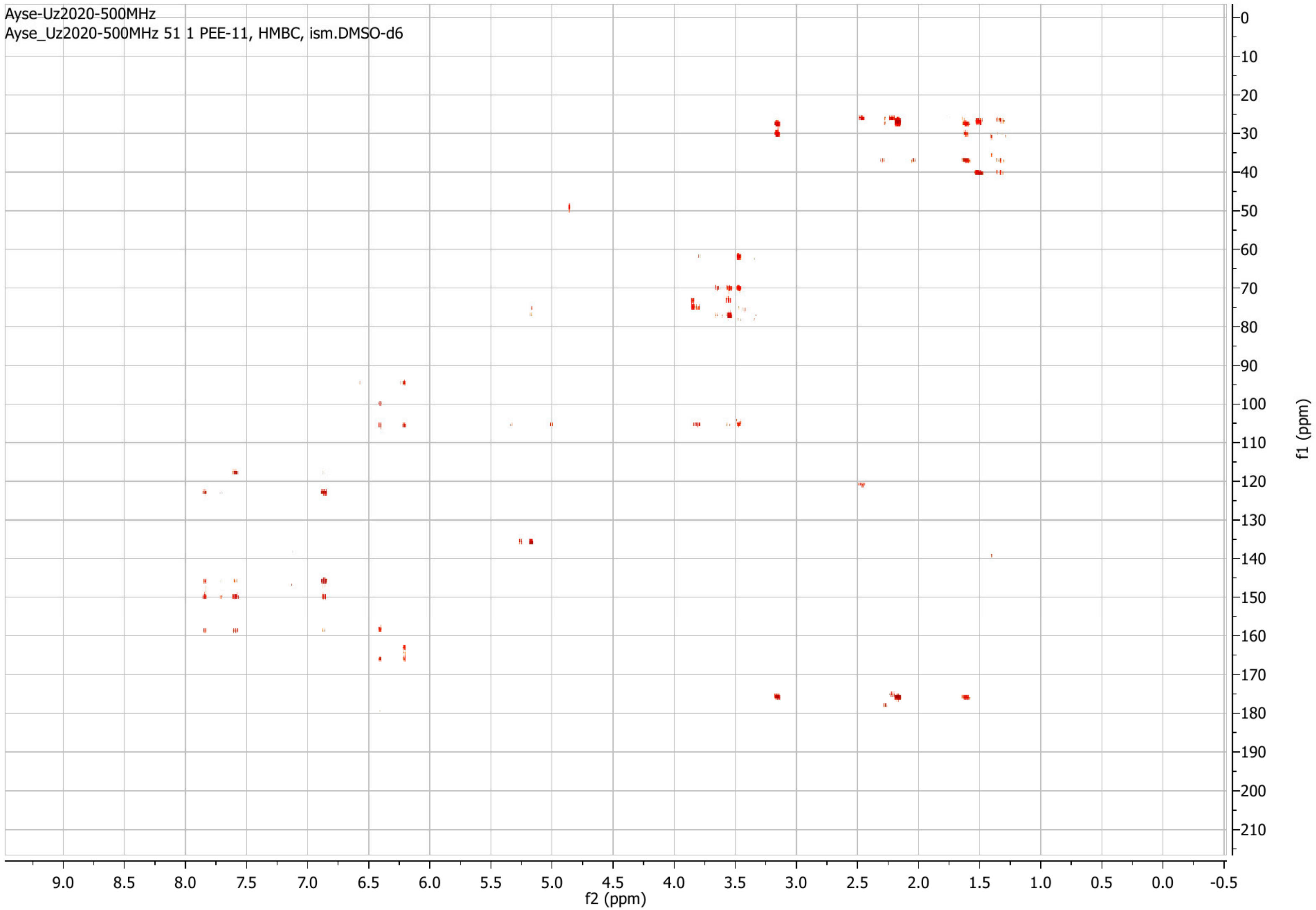

Key long-range correlation: Long-range cross-peaks from the anomeric proton region (δH approximately 5.17–5.26) to the oxygenated quercetin C-3 region support 3-O-glycosylation. The shared aglycone correlations and extensive overlap are consistent with an inseparable mixture of the glucose and galactose epimers rather than two independently purified samples.

Figure S22. Positive-ion full-scan ESI mass spectrum of the PEE-4a/PEE-4b mixture (internal raw-data code PEE-11).

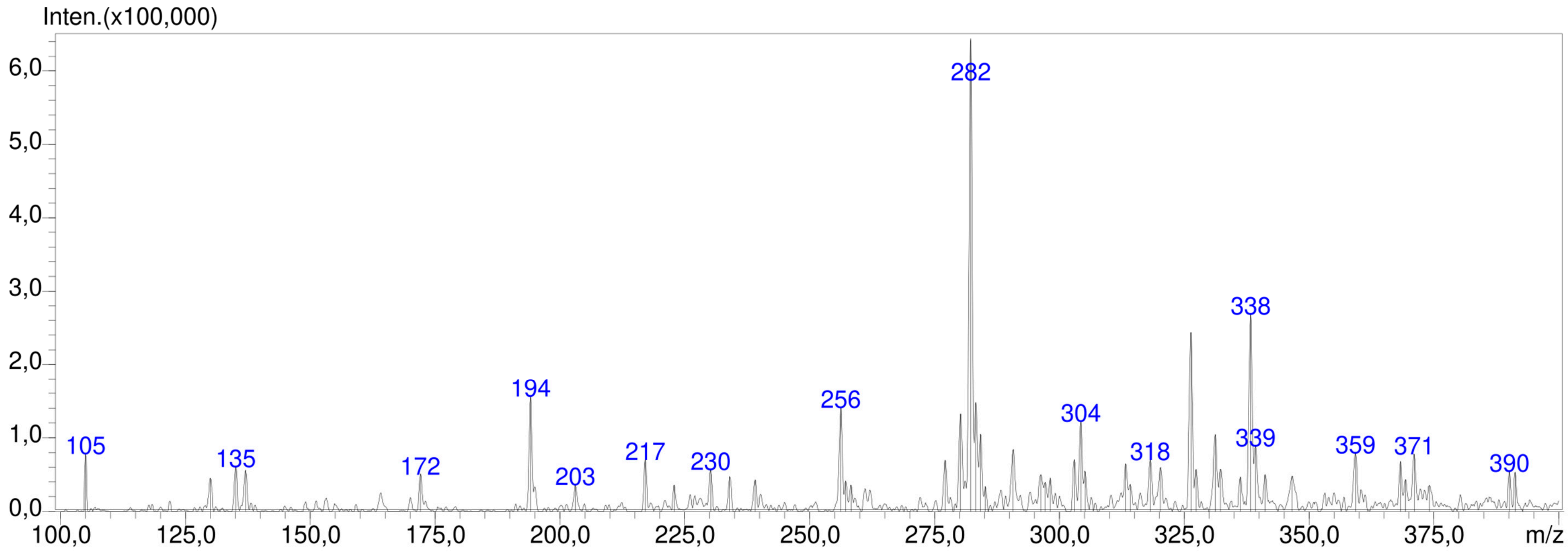

Interpretive limitation: An ion at m/z 203 was consistent with a sodiated hexose-related ion (calcd for $C_6H_{12}NaO_6^+$, 203.0526), while a nominal ion at m/z 304 was observed in the quercetin-aglycone mass region. Because the scan did not include an unequivocal intact glycoside ion and no precursor-specific MS/MS experiment was performed, these signals were considered supportive only and were not used for definitive structural assignment. Isoquercitrin and hyperoside have the same molecular formula ($C_{21}H_{20}O_{12}$) and cannot be differentiated by molecular mass alone.
